# Deep reinforcement learning navigation via decision transformer in autonomous driving

**DOI:** 10.3389/fnbot.2024.1338189

**Published:** 2024-03-19

**Authors:** Lun Ge, Xiaoguang Zhou, Yongqiang Li, Yongcong Wang

**Affiliations:** ^1^School of Modern Post (School of Automation), Beijing University of Posts and Telecommunications, Beijing, China; ^2^Mogo Auto Intelligence and Telematics Information Technology Co., Ltd, Beijing, China; ^3^Neolix Technologies Co., Ltd, Beijing, China

**Keywords:** autonomous driving, deep reinforcement learning (DRL), Soft Actor-Critic (SAC), variational autoencoder (VAE), Partially Observable Markov Decision Processes (POMDPs), multimodal state space

## Abstract

In real-world scenarios, making navigation decisions for autonomous driving involves a sequential set of steps. These judgments are made based on partial observations of the environment, while the underlying model of the environment remains unknown. A prevalent method for resolving such issues is reinforcement learning, in which the agent acquires knowledge through a succession of rewards in addition to fragmentary and noisy observations. This study introduces an algorithm named deep reinforcement learning navigation via decision transformer (DRLNDT) to address the challenge of enhancing the decision-making capabilities of autonomous vehicles operating in partially observable urban environments. The DRLNDT framework is built around the Soft Actor-Critic (SAC) algorithm. DRLNDT utilizes Transformer neural networks to effectively model the temporal dependencies in observations and actions. This approach aids in mitigating judgment errors that may arise due to sensor noise or occlusion within a given state. The process of extracting latent vectors from high-quality images involves the utilization of a variational autoencoder (VAE). This technique effectively reduces the dimensionality of the state space, resulting in enhanced training efficiency. The multimodal state space consists of vector states, including velocity and position, which the vehicle's intrinsic sensors can readily obtain. Additionally, latent vectors derived from high-quality images are incorporated to facilitate the Agent's assessment of the present trajectory. Experiments demonstrate that DRLNDT may achieve a superior optimal policy without prior knowledge of the environment, detailed maps, or routing assistance, surpassing the baseline technique and other policy methods that lack historical data.

## 1 Introduction

The automobile industry has increasingly prioritized autonomous (González et al., 2015) driving technology due to the ongoing advancements in science and technology. The implementation of driverless vehicles heavily relies on integrating an autonomous driving navigation system, a fundamental component. Analyzing environmental data enables autonomous driving navigation using numerous sensors and algorithms. The utilization of machine learning enables the application of learning-based techniques in making autonomous driving decisions. Imitation learning is often regarded as the prevailing approach when driving regulations are acquired automatically through the analysis of expert driving data. Nevertheless, imitation learning is not without its limitations. Firstly, acquiring substantial quantities of authentic, contemporaneous driving data from proficient experts is a prerequisite, a process that might incur significant costs and consume considerable time. Furthermore, the limited learning capacity of the system restricts its ability to acquire driving skills beyond those displayed in the dataset. Consequently, this limitation may give rise to safety concerns as the system may not possess the necessary knowledge to handle hazardous scenarios not encompassed within the dataset. Thirdly, it is improbable that the imitation learning strategy may surpass human performance, given that the human driving expert assumes the role of a learning supervisor. Given these constraints, it is imperative to investigate alternative methodologies for decision-making in autonomous driving. One such method is reinforcement learning, which automatically enhances and discovers new policies without manual design.

In autonomous driving navigation, reinforcement learning (Kiran et al., 2021; Ye et al., 2021) can help vehicles learn optimal navigation policies by interacting with the road environment. Through continuous trial and error and reward mechanisms, reinforcement learning algorithms can enable vehicles to gradually learn to deal with various complex traffic situations and road conditions. Establishing a suitable reward system is of utmost importance in the context of reinforcement learning for self-driving navigation (Morales et al., 2021). Reinforcement learning algorithms can effectively guide the vehicle to acquire appropriate behavior by employing positive rewards, such as completing the navigation job, or negative rewards, such as contravening traffic regulations. The field of autonomous driving is advancing quickly, with reinforcement learning showing promise in enabling agents to learn how to drive without relying on expert data or manual design. This method entails the agent learning to make decisions in various scenarios, including hazardous ones, potentially surpassing the skills of even the most experienced human drivers. By harnessing the power of reinforcement learning, autonomous driving systems can become more sophisticated and better equipped to handle the intricacies of real-world driving situations.

Nevertheless, implementing reinforcement learning in autonomous driving navigation has certain hurdles. Training (Kaelbling et al., 1998) effective policies is a formidable task primarily because of the intricacies associated with the infinite-dimensional space. Furthermore, complicated and uncertain road environments further compound the challenges in making navigation decisions. The substantial quantity of necessary exploration impedes the practical implementation of large action spaces. This circumstance will result in unsatisfactory outcomes of reinforcement learning-driven policy learning for complex real-world tasks. The occlusion and noise experienced by the sensors hinder the Agent's capacity to perceive the actual status of the surroundings accurately. The Agent cannot reach an optimal conclusion given the existing situation, which is untrue. Most current methodologies employ front-view images as input for end-to-end learning policies. This methodology results in highly complex and dimensional visual characteristics. Another study area that deserves attention is the application of deep reinforcement learning in autonomous Driving. Using elementary deep reinforcement learning methods, such as DQN (Mnih et al., 2013, 2015), may provide limitations in addressing intricate navigation challenges. In recent times, there has been notable progress in developing deep reinforcement learning algorithms with increased efficacy. Autonomous driving technology has limitations that restrict its use to only a few tasks.

This study introduces a novel technique called deep reinforcement learning navigation via decision transformer (DRLNDT). The Transformer model uses the Soft Actor-Critic approach to gain accurate information about the present state by considering the past trajectory state. This method helps the Agent avoid misinterpretations or incorrect judgments regarding the surroundings, possibly due to sensor occlusion or noise. The conventional reinforcement learning model is constructed within the Markov Decision Process (MDP) framework. Our methodological approach is based on the Partially Observable Markov Decision Process (POMDP). The data collected (Ghosh et al., 2021) by the Agent's sensor may need to be more accurate as it depends on a hidden variable that existed in the past state of the sensor and may not accurately represent the current environmental conditions. High-quality images are crucial for capturing a complete and accurate representation of reality and extracting valuable information. Because images of high-quality and larger dimensions can more precisely depict the real world, providing a more comprehensive range of valuable data. Nevertheless, utilizing high-resolution images (Nair et al., 2015; Andrychowicz et al., 2020; Janner et al., 2021) containing intricate visual features results in the intricacy of sample learning and the occupation of substantial memory space, resulting in ineffective learning and inadequate algorithm training. This study uses a variational autoencoder (VAE) to extract latent vectors from high-resolution photos. These latent vectors are then substituted for the original high-resolution images, reducing dimensionality while preserving the salient features of the samples to the greatest extent possible. In addition, we utilize tricks of the Soft Actor-Critic (SAC) policy, including changing temperature and variable learning rate, among others, to enhance the algorithm's efficacy. The conclusive experimental findings demonstrate that our method outperforms the baseline algorithm.

In this paper, we provide the following contributions:

1. In this study, we provide a novel algorithm named deep reinforcement learning navigation via decision transformer (DRLNDT), which leverages a transformer model to acquire knowledge of the current state based on past states. The primary objective of this approach is to mitigate judgment errors that arise due to sensor noise or occlusion in a singular state.
2. The variational autoencoder (VAE) extracts latent vectors from high-quality images, reducing the dimensionality of the state space while preserving essential image properties. In conclusion, optimizing image memory allocation has improved training efficiency and outcomes.
3. The method enables an autonomous vehicle to navigate visually from its starting point to its destination without relying on route direction or high-precision maps, utilizing only high-quality monocular raw photos and producing successful outcomes.
4. Our study incorporates vector states such as velocity and position, which can be effortlessly obtained from the vehicle's intrinsic sensors. Furthermore, we introduce latent vectors from high-quality images to construct a multimodal state space. This method enables the agents to evaluate the current trajectory based on the states, leading to improved overall performance outcomes.

This paper is organized into several sections, each with a specific focus. Section 2 of this paper focuses on the elucidation and explication of pertinent research in the field of autonomous driving. The text emphasizes reinforcement learning techniques used in autonomous driving and the approaches to address POMDPs through reinforcement learning algorithms. Section 3 of this paper introduces various forms and definitions intended to facilitate the comprehension and contextualization of the content. This particular section holds significant importance as it establishes the fundamental basis for the methodology put forth in Section 4. Section 4 of this paper introduces the DRLNDT algorithm, which serves as the central focus of the study and encompasses the most intricate technical aspects. Section 5 depicts the experimental outcomes obtained by implementing our algorithm on the CARLA platform. The results substantiate the superiority of our approach over the baseline approach. The available evidence adequately supports the efficacy of our approach. In conclusion, Section 6 summarizes the essential findings and offers suggestions for future research directions.

## 2 Related works

We reviewed recent literature on “Reinforcement learning-based autonomous driving” and “Deep reinforcement learning for POMDPs,” summarizing their research.

### 2.1 Reinforcement learning-based autonomous driving

Kendall et al. (2019) demonstrated the application of deep reinforcement learning to autonomous driving, where a model uses a single monocular image as input to learn a lane following policy. The model is trained through several rounds with randomly initialized parameters. The reward is the distance the vehicle travels without the driver's intervention. The approach relies on continuous, model-free deep reinforcement learning, with all exploration and optimization taking place in the vehicle.

Chen et al. (2019) and his team have developed a framework for deep reinforcement learning in urban autonomous driving scenarios. The framework uses a bird's-eye view and visual coding to capture low-dimensional latent states. The team implemented several state-of-the-art model-free deep RL algorithms in the framework and improved their performance. They tested the performance of the framework in the challenging task of navigating a circular intersection with dense surrounding vehicles and found that it performed excellently compared to the baseline. Additionally, the team introduced and tested three model-free deep RL algorithms to evaluate their success rate in the roundabout intersection task. The results demonstrate the effectiveness of the proposed framework and algorithms in solving complex urban driving tasks.

Liang et al. (2018) present a new Controllable Imitation Reinforcement Learning (CIRL) model for DRL-based autonomous vehicle driving in a high-fidelity vehicle fidelity simulator. CIRL combines Controllable Imitation Learning with DDPG policy learning to address sample inefficiency in reinforcement learning. It outperforms previous approaches, achieving state-of-the-art driving performance on the CARLA benchmark. The CIRL model optimizes the policy network with specialized steering angle rewards for targeting different driving scenarios. It has excellent generalization capabilities across various environments and conditions.

Anzalone et al. (2022) propose a reinforcement curriculum learning method for training agents in a driving simulation environment. The Agent has two phases of training. In the first phase, it starts from a fixed location and drives according to the speed limit without any traffic. In the second phase, the Agent encounters diverse starting locations and randomly generated pedestrians. The driving policy is evaluated quantitatively and qualitatively.

Ozturk et al. (2021) propose the use of curriculum reinforcement learning for autonomous driving in different road and weather conditions. This study tackled the challenge of tuning Agents for optimal performance and generalization in various driving scenarios by using curriculum reinforcement learning. Results showed significant improvement in performance and a reduction in sample complexity. Different courses provided different benefits, indicating potential for future research in automated curriculum training.

Yeom (2022) propose a deep reinforcement learning (DRL) based collision-free path planning architecture for mobile robots, which can navigate unknown environments without supervision. The architecture uses DRL to figure out the unknown environment and predicts control parameters for the mobile robots in the next time step. Experimental results show that the proposed architecture can successfully solve complex navigation problems in dynamic environments.

We found that although all of these studies had some achievements, they did not achieve the task of navigating from the initial position to the termination position. Forward-looking images were used in some methods, but most were low-resolution for algorithm convenience. High-quality images are necessary for more features and real-world applications. Most navigation agents use routing, which the original project did not intend. Routing guides the optimal policy but is not always optimal. Computation needs a high-precision map, which increases costs. It goes against the original project's idea of minimizing the need for high-precision maps.

### 2.2 Deep reinforcement learning for POMDPs

Heess et al. (2015) used neural networks to solve continuous control problems, and the method was successful in fully observed states. Control problems in real-world scenarios are often only partially observed due to various factors, such as sensor limitations, changes in the controlled object that go unnoticed, or state aliasing caused by function approximation. This article proposes the use of recurrent neural networks trained with temporal backpropagation in model-free continuous control algorithms to tackle partially observed domains.

Igl et al. (2018) proposed a method called Deep Variational Reinforcement Learning (DVRL) to address the challenges of partially observable sequential decision problems. This method helps the agent learn a generative model of the environment and efficiently aggregate information. Researchers developed an n-step approximation of ELBO and a policy for the control task. DVRL outperforms previous approaches and accurately approximates the confidence distribution on latent states. Additionally, a second RNN summarizes the set of particles, accounting for the uncertainty of the latent state after the following action.

Zhu et al. (2017) proposed a new method called Action Specific Deep Recurrent Q Network (ADRQN) to improve the learning performance in partially observable domains. This proposed method encodes actions with a multilayer perceptron (MLP) and combines them with observation features from a convolutional neural network (CNN) to create action-observation pairs. These pairs generate a time series integrated by a Long Short-Term Memory (LSTM) layer to infer latent states. A fully connected layer computes the *Q*-value, predicting expected rewards for actions in a given state. Tested in partially observable domains, including Atari, this method outperformed state-of-the-art methods.

Hausknecht and Stone (2015) replaced the first fully connected layer of a Deep Q Network (DQN) with an LSTM to add loops and investigated its effect. DRQN, a Deep Recurrent Q Network, integrates temporal information and performs as well as DQN on a standard and partially observed Atari game. Its performance varies with observability and degrades less than DQN when evaluated with partial observations. Looping is a viable alternative to stacking frame histories in the DQN input layer and adapts better to changes in observation quality when evaluated. However, looping does not provide any systematic benefits over stacking observations in the input layer of a convolutional network for non-flickering games.

Chen et al. (2021) use transformer to model high-dimensional distributions of semantic concepts and their latent application to sequential decision problems formalized as reinforcement learning (RL). A new approach for Reinforcement Learning (RL) policy training has been proposed, which uses sequential modeling objectives to train Transformer models with experience data. The architecture, called Decision Transformer, can transform RL problems into conditional sequence modeling. It has been shown to perform well on Atari, OpenAI Gym, and Key-to-Door tasks.

These methods are very inspiring, and we have proposed the DRLNDT method to enable autonomous driving navigation. The transformer model is utilized to learn the actual state from the historical data, thus reducing decision errors caused by object occlusion or sensor noise. The results of our method are better than those of the Baseline method in CARLA.

## 3 Backgrounds

The Partially Observable Markov Decision Process (POMDP) is a type of sequential decision-making problem that involves modeling the environment based on its location while also considering incomplete and noisy observations. This paper presents a novel approach known as deep reinforcement learning navigation via decision transformer (DRLNDT). The proposed method incorporates a Decision Transformer, which learns the state based on past observations. It then utilizes this learned information to guide the Agent in navigating the task, following a reward learning scheme, from the initial to the termination position. Variable Autoencoder (VAE) (Loaiza-Ganem and Cunningham, 2019; Wei et al., 2020) is a neural network type that can learn a compressed representation of input data by encoding it into the status space and decoding it back into the original space. This paper employs the variational autoencoder (VAE) to enhance the algorithm's performance. DRLNDT utilizes a Transformer neural network architecture to capture temporal dependencies within observations and actions effectively. This capability enhances self-driving vehicles' decision-making process in partially observable urban environments. The paper introduces the Transformer algorithm, integrated with a reinforcement learning algorithm. The reinforcement learning algorithm employs a variational autoencoder (VAE) for compressive characterization of the image data. Next, integrating multimodal observations' time series is performed using the Transformer model. The latent state is acquired by the layer, which subsequently employs the fully connected layer to estimate the value and policy functions, similar to standard reinforcement learning algorithms.

### 3.1 Markov decision processes

A sequential decision (Arulkumaran et al., 2017) problem refers to a scenario in which an agent is tasked with making a sequence of decisions over time, where each decision's outcome impacts the subsequent decisions. In these types of problems, it is common for the Agent to possess knowledge of the dynamic model of the environment, which implies that the Agent has access to information regarding how the environment will change in response to its actions. In order to establish a formal framework for addressing these issues, researchers employ a mathematical construct known as a Markov Decision Process (MDP) (Puterman, 2014), defined by a 4-tuple denoted as < *S, A, P, R* >. Here. *S* represents the set of all possible states in the environment, *A* represents the set of possible actions the Agent can take, *P* represents the probability distribution of the next state given the current state and action, and *R* represents the mapped reward function where each state-action pair is rewarded with a scalar value. During each iteration, the Agent makes a decision by selecting an action *a*_*t*_ from a set of possible actions *A*, based on the current state *s*_*t*_ from a set of possible states *S*, and its policy π which maps states to actions. As a consequence of this action, the Agent receives an immediate reward *r*_*t*_ that is drawn from a distribution *R*(*s*_*t*_, *a*_*t*_). Additionally, the Agent transitions to a new state *s*_*t*+1_, which is sampled from the probability distribution *P*(*s*_*t*+1_|*s*_*t*_, *a*_*t*_). The policy π(*a*_*t*_|*s*_*t*_) is utilized to calculate the state and state-action marginals, which are represented as ρ_π_(*s*_*t*_) and ρ_π_(*s*_*t*_, *a*_*t*_), respectively. The margins in question denote the likelihood of being in a specific state or state-action pair under the policy denoted as π. The objective of reinforcement learning is to identify the optimal policy that maximizes the expected discounted reward *R*_*t*_. The discount factor γ, which falls within the range of [0, 1], determines the relative significance of immediate rewards compared to future rewards.


(1) $$\begin{array}{l} R_{t}=r_{t}+\gamma r_{t+1}+\gamma^{2}r_{t+2}+\ldots \end{array}$$

The discount rewards are computed using Equation (1), which provides a concise representation of the rewards acquired at each time, considering the discount factor associated with each timestep. In the context of Markov Decision Processes (MDPs), the determination of the optimal policy can be achieved through the process of value iteration. This iterative procedure entails the updating of the value function, which serves as a representation of the expected discounted reward for each state given a specific policy.

### 3.2 Soft Actor Critic

The *Q*-learning (Watkins and Dayan, 1992) technique was introduced by Watkins and Dayan in 1992 as a solution to reinforcement learning problems characterized by unknown environmental dynamics. The technique is considered model-free as it does not necessitate prior knowledge of the environment or its dynamics. *Q*-learning aims to estimate the value associated with executing an action and adhering to an optimal policy π within a specific state. The quantity above is commonly referred to as the state-action value or, more succinctly, the *Q*-value. The *Q*-value measures the anticipated total reward achieved by selecting a specific action in a given state and adhering to the optimal policy. The *Q*-value is defined recursively as the summation of the immediate reward acquired from the action and the discounted value of the subsequent state-action pair. The utilization of a discount factor γ, which falls within the range of [0, 1], serves the purpose of discounting future rewards and facilitating the convergence of *Q* values. The optimal policy π^*^ can be derived by selecting the action with the maximum *Q*-value in every state. *Q*-learning is an algorithm that operates off-policy, meaning it learns the *Q*-value of a target policy while adhering to various behavioral policies.

The function *Q*_π_(*s, a*) is a formal mathematical representation denoting the expected collect reward that an Agent obtains when it selects action *a* within state *s* and adheres to policy π. The *Q*-value is acquired through an iterative process, wherein the Agent continually updates its estimate of the *Q*-value by considering the rewards it obtains during its interactions with the environment.


(2) $$\begin{array}{l} Q_{\pi }\left(s,a\right)=E_{\pi }\left(R_{t}|s_{t}=s,a_{t}=a\right) \end{array}$$

The Equation (2) represents the anticipated reward that the Agent is expected to obtain at a given time *t*, under the condition that the Agent is in a specific state denoted as *s* and selects a particular action denoted as *a*, by the policy denoted as π. *Q*-values play a crucial role in reinforcement learning, enabling the Agent to make optimal action selections within a specific state. The Agent selects the action with the highest *Q* value within the given state. The process of updating *Q*-values can be accomplished through the utilization of a method known as *Q* learning. This technique entails the modification of the *Q*-value associated with the present state-action pair by considering the highest *Q*-value among the subsequent state-action pairs. The *Q*-learning algorithm is classified as an off-policy method, which implies that the Agent can learn the optimal *Q*-value even when it adheres to a policy that differs from the one being evaluated. The *Q*-value is employed for approximating the value of the policy, specifically the anticipated cumulative reward that the Agent will obtain by adhering to the policy. The maximization of the value function determines the optimal policy.

The *Q* function is a mathematical function that provides an estimation of the expected future reward when a specific action is taken within a specific state in Equation (3).


(3) $$\begin{array}{l} Q\left(s,a\right)=Q\left(s,a\right)+\beta \left(r+\gamma {\underset{a}{max}}^{\prime }Q\left(s^{\prime },a^{\prime }\right)-Q\left(s,a\right)\right) \end{array}$$

The equation presented herein represents the *Q*-learning update rule, which is a fundamental element of numerous reinforcement learning algorithms. The equation incorporates various components, namely the current state (*s*), the action taken (*a*), the reward received (*r*), the subsequent state (*s*′), and a discount factor (γ). This equation updates the *Q* value of the current state-action pair by adding the scaling difference between the estimated *Q* value of the following state-action pair and the *Q* value of the current state-action pair. The scaling factor β is the learning rate, which determines how much new information is incorporated into existing estimates. In instances with many states where it is impossible to save *Q* values for all state-action combinations, the equation above is used. In contrast, a function approximator, such as a neural network, estimates the *Q* values for previously unobserved state-action combinations. The DQN method illustrates a reinforcement learning methodology that utilizes a neural network to estimate *Q* values. Using the current state and action as input variables, the neural network, identified by the parameter θ, generates an estimated value *Q* for a specific state-activity combination.

In contrast to the DQN algorithm, Soft Actor-Critic (SAC) (Haarnoja et al., 2018) is an off-policy Actor-Critic algorithm that operates within a maximum entropy reinforcement learning framework. The primary objective of SAC is to optimize both the expected return and entropy. SAC contains several modifications to accelerate training and enhance the stability of hyperparameters, such as automatic tuning of the constraint formulas for the temperature hyperparameter. The maximum entropy objective extends the conventional aim employed in conventional reinforcement learning methods. Adding an entropy element to the objective signifies that the optimal policy seeks to maximize its entropy at each accessed state.


(4) $$\begin{array}{l} \pi^{*}=\underset{\pi }{argmax}\;\underset{t}{\sum }E_{\left(s_{t},a_{t}\right)\sim \rho_{\pi }}\left[r\left(s_{t},a_{t}\right)+\alpha H\left(\pi \left(\cdot |s_{t}\right)\right)\right] \end{array}$$

Maximizing the expected reward and entropy of each state determines the optimal policy. The parameter α in the Equation (4) governs the relative significance of the entropy term concerning the reward, influencing the optimal policy's probabilistic characteristics. The maximum entropy aim applies when the best policy necessitates randomization or stochasticity, such as in exploration tasks or when confronted with unpredictable settings. The discount factor, denoted as γ, is a scalar within the range of 0 to 1, which plays a crucial role in determining the relative significance of future rewards within the context of decision-making. A discount factor of zero implies that only incentives in the present period are considered. In contrast, a discount factor of one indicates that rewards in the future are given equal importance to immediate rewards. The discount factor is crucial in infinite horizon problems since it guarantees the convergence of the expected reward and entropy to a limited value. With the discount factor, the cumulative value of predicted rewards and entropy may remain the same toward infinity, making the objective function's optimization attainable. Incorporating the discount factor enables the algorithm to effectively weigh the significance of immediate benefits against those obtained in the future, facilitating more optimal decision-making over extended time horizons.

Determine the solution for the optimal *Q*-function, which establishes a correspondence between a state-action pair and a value that denotes the anticipated long-term benefit associated with executing that action in that state and afterward adhering to the optimal policy. From the ideal *Q*-function, one can deduce the best policy. The suggested algorithm is a Soft Actor-Critic (SAC) approach that is formulated using the policy iteration framework. The *Q*-function associated with the present policy is assessed, and the policy is then modified through the utilization of off-policy gradient updating. Off-policy suggests a difference between the policy being updated and the policy that produced the data for modification. The Maximum Entropy Reinforcement Learning framework serves as the foundation for the Soft Actor-Critic algorithm, where the Actor's objective is to maximize predicted reward and entropy.

Soft policy iteration is a generalized algorithm for learning optimal maximum entropy policies. The algorithm alternates between policy evaluation and policy improvement in a maximum entropy framework. The derivation of the algorithm is based on a tabular setup that allows for theoretical analysis and convergence guarantees. The algorithm aims to converge to the optimal policy among a set of strategies, which may correspond to a set of parameterized densities. The set of strategies to which the algorithm converges is not fixed and can vary depending on the specific problem to be solved. The algorithm aims to maximize the expected return while maximizing the entropy of the strategies. The entropy of a policy is a measure of the stochasticity of the policy, and maximizing it encourages exploration and prevents the policy from falling into a local optimum. The algorithm is called “Soft” because it uses a Soft-valued function instead of a hard-valued function. Soft-valued functions are smoothed versions of hard-valued functions, which are easier to optimize and prevent overfitting.

Soft policy iteration is a technique employed in the field of reinforcement learning to assess the efficacy of a policy and determine its worth by optimizing the maximum entropy target. During the policy evaluation phase of Soft policy iteration, it is possible to calculate Soft *Q* values for fixed policy iterations. The computation of the Soft *Q* value involves the iterative use of the modified Bellman backup operator, denoted as *T*^π^ in Equation (5).


(5) $$\begin{array}{l} T^{\pi }Q\left(s_{t},a_{t}\right)≜r\left(s_{t},a_{t}\right)+\gamma E_{s_{t+1}\sim p}\left[V\left(s_{t+1}\right)\right] \end{array}$$

where *r*(*s*_*t*_, *a*_*t*_) represents the reward obtained for taking an action *a*_*t*_ in state *s*_*t*_, γ represents the discount factor, and *p* represents the transfer probability distribution. The Soft *Q*-value is calculated using the Soft state value function *V*(*s*_*t*_) in Equation (6).


(6) $$\begin{array}{l} V\left(s_{t}\right)=E_{a_{t}\sim \pi }\left[Q\left(s_{t},a_{t}\right)-\alpha log\pi \left(a_{t}|s_{t}\right)\right] \end{array}$$

The value of *Q* for taking an action *a*_*t*_ in state *s*_*t*_ is denoted as *Q*(*s*_*t*_, *a*_*t*_). The probability of taking an action in state *s*_*t*_ according to the policy π is represented as π(*a*_*t*_|*s*_*t*_). The temperature parameter α regulates the balance between maximizing the expected payoff of the policy and maximizing the entropy. By repeatedly applying the Bellman backup operator *T* to any initial *Q* function *Q* : *S* × *A* → *R*, one can obtain the Soft *Q* function for any policy π. The Soft *Q* function is an advantageous instrument for assessing policies in the context of reinforcement learning due to its consideration of policy uncertainty and promotion of exploration.

To create a feasible approximation of the Soft policy iteration, a function approximator can be utilized for the Soft *Q* function and policy. Instead of assessing and enhancing the convergence aspect, it is suggested to employ stochastic gradient descent as an alternative approach to optimize both networks simultaneously. The Soft *Q* function and policy are parameterized by a neural network with θ and *phi* parameters. The Soft *Q* function can be modeled as an expressive neural network. In contrast, the policy can be modeled as a Gaussian function, with the neural network providing the mean and covariance. The rules for updating these parameter vectors are subsequently derived and employed to optimize the network during the training process. The objective is to tackle the issues of significant sample complexity and vulnerability to hyperparameters commonly observed in model-free deep reinforcement learning methods through the utilization of function approximators and stochastic gradient descent. The suggested methodology is grounded in the framework of maximum entropy reinforcement learning. This paradigm seeks to optimize both the expected return and entropy, enabling the Agent to accomplish the goal while exhibiting a high degree of randomness in its actions.

The Soft *Q* function is a modified version of the *Q* function employed in the field of reinforcement learning, which integrates a component of entropy to promote exploration. The parameters of the Soft *Q* function are optimized through training in order to minimize the Soft Bellman residual, which serves as a metric for quantifying the discrepancy between the anticipated *Q* value and the real *Q* value.


(7) $$\begin{array}{l} J_{Q}(\theta )=E_{(s_{t},a_{t})\sim D}[\frac{1}{2}(Q_{\theta }(s_{t},a_{t})-(r(s_{t},a_{t})\\ \;+\gamma E_{s_{t+1}\sim p}[V_{\bar{\theta }}(s_{t+1})]){)}^{2}] \end{array}$$

The Soft Bellman residual is formally defined in Equation (7), whereby it encompasses the calculation of the expected value of the squared discrepancy between the predicted *Q*-value and the summation of the reward and subsequent state discount values. The utilization of the Soft *Q* function argument serves as an implicit parameterization of the value function, as specified in Equation (6). A crucial component of the SAC algorithm, the value function estimates the expected reward for a given state. The parameters of the Soft *Q* function are optimized by the utilization of stochastic gradient descent, a widely employed optimization method within the field of deep learning. The optimization of the Soft *Q* function parameters is a crucial component of the SAC method as it enables the Agent to acquire a precise estimation of the anticipated reward associated with a specific state-action combination. By reducing the residuals of the Bellman Soft equation, the Agent can acquire improved decision-making abilities and attain enhanced performance across a range of reinforcement learning challenges.


(8) $$\begin{array}{l} {\hat{\nabla }}_{\theta }J_{Q}(\theta )=\nabla_{\theta }Q_{\theta }(a_{t},s_{t})(Q_{\theta }(s_{t},a_{t})-(r(s_{t},a_{t})+\gamma (Q_{\bar{\theta }}(s_{t+1},a_{t+1})\\ \;-\alpha log(\pi_{\phi }(a_{t+1}|s_{t+1})))) \end{array}$$

The Soft *Q* function arguments, which are obtained from Equation (6), implicitly parameterize the value function. The objective is optimized using stochastic gradient descent, where the stochastic gradient is computed using the gradient of the *Q* function with respect to its parameters in Equation (8). The *Q* function is a mathematical function that accepts the current state (*s*_*t*_) and action (*a*_*t*_) as its input and produces the anticipated reward for that specific state-activity combination. The expected reward is equal to the sum of the instantaneous reward (*r*(*s*_*t*_, *a*_*t*_)) and the discounted expected reward (*Q*_θ_(*s*_*t*+1_, *a*_*t*+1_)) for the next state-action pair. The Soft *Q* function is modified by the addition of a term that promotes exploration, which is determined by the temperature parameter α and the policy function π_ϕ_(*a*_*t*_ + 1|*s*_*t*_ + 1). The update also employs a target Soft *Q* function with parameter $\bar{\theta }$, which is derived as an exponentially shifted mean of the Soft *Q* function weights. The utilization of this target *Q* function serves the purpose of stabilizing the training process and mitigating the occurrence of overfitting.


(9) $$\begin{array}{l} J_{\pi }\left(\phi \right)=E_{s_{t}\sim D}\left[E_{a_{t}\sim \pi_{\phi }}\left[\alpha log\left(\pi_{\phi }\left(a_{t}|s_{t}\right)\right)-Q_{\theta }\left(s_{t},s_{t}\right)\right]\right] \end{array}$$

Equation (9) denotes the goal function *J*_π_(ϕ) employed for the purpose of acquiring the policy parameters in the Soft Actor-Critic (SAC) algorithm. The objective function *J*_π_(ϕ) entails the maximization of the expected payout and entropy of the Actor while executing the job. There exist other alternatives for minimizing the objective function *J*_π_. However, in the context of Soft Actor-Critic (SAC), the reparameterization method is employed as a means to attain a reduced variance estimator. The reparameterization technique entails converting the random variables used to sample the actions from the policy into noise variables and differentiable functions of the policy parameters, thereby permitting the gradient to be back-propagated through a network of policy and target densities, which in SAC are *Q*-functions represented by neural networks. The utilization of the reparameterization methodology yields an estimator with reduced variance in comparison to the likelihood ratio gradient estimator commonly employed in policy gradient methods.

SAC employs a reparameterization technique for neural network restructuring. The equation *a*_*t*_ = *f*_ϕ_(ϵ_*t*_; *s*_*t*_) is utilized to establish a mapping between states and actions within the context of a reinforcement learning problem. The function *f*_ϕ_(ϵ_*t*_; *s*_*t*_) represents the transformation of the neural network, with ϕ denoting the parameters of the network. The input to the neural network transformation is the noise vector ϵ_*t*_, which is sampled from a stationary distribution, such as a spherical Gaussian distribution. The utilization of noise vectors as inputs serves the objective of introducing stochasticity into the policy, hence facilitating the Agent's exploration of the environment and enhancing its ability to acquire more effective methods. The outcome of the neural network transformation corresponds to the action executed by the Agent in reaction to the present state *s*_*t*_. By employing this particular reparameterization technique, the SAC algorithm is capable of acquiring policies that are more versatile and articulate, hence enabling them to be adjusted to various scenarios within the environment.


(10) $$\begin{array}{l} J_{\pi }\left(\phi \right)=E_{s_{t}\sim D,\epsilon_{t}\sim N}\left[\alpha log\pi_{\phi }\left(f_{\phi }\left(\epsilon_{t};s_{t}\right)|s_{t}\right)-Q_{\theta }\left(s_{t},f_{\phi }\left(\epsilon_{t};s_{t}\right)\right)\right] \end{array}$$

Equation (10) is an altered variant of Equation (9) that includes an implicit definition of the Actor policy π_ϕ_ based on the function *f*_ϕ_. A neural network called *f*_ϕ_ is a function that links actions to current states and timesteps. The objective in Equation (10) is expressed as a mathematical function that depends on two variables: the policy parameter ϕ and the *Q*-value parameter θ. The objective function in Equation (10) is estimated by combining data *D* in the replay buffer with noise *N* derived from a normal distribution. The expression αlogπ_ϕ_(*f*_ϕ_(ϵ_*t*_; *s*_*t*_)|*s*_*t*_) in Equation (10) denotes the entropy of the policy, which promotes exploration and stochasticity in the behavior of the Actor. Equation (9) introduces the notation *Q*_θ_(*s*_*t*_, *f*_ϕ_(ϵ_*t*_; *s*_*t*_)|*s*_*t*_), which denotes the *Q*-value of the Critic. This *Q*-value serves as a metric for estimating the anticipated rewards associated with the current condition and action. The gradient approximation formula is represented as ${\hat{\nabla }}_{\phi }J_{\pi }\left(\phi \right)$, which serves as an unbiased estimator of the gradient in Equation (11).


(11) $$\begin{array}{l} {\hat{\nabla }}_{\phi }J_{\pi }(\phi )=\nabla_{\phi }\alpha log(\pi_{\phi }(a_{t}|s_{t}))+(\nabla_{a_{t}}\alpha log(\pi_{\phi }(a_{t}|s_{t}))\\ \;-\nabla_{a_{t}}Q(s_{t},a_{t}))\nabla_{\phi }f_{\phi }(\epsilon_{t};s_{t}) \end{array}$$

The gradient estimator extends the DDPG (Silver et al., 2014) policy gradient to any easily handled stochastic policy. The formula involves evaluating *a*_*t*_ at *f*_ϕ_(ϵ_*t*_; *s*_*t*_). The gradient estimator involves two terms, the first of which is ∇_ϕ_α*log*(π_ϕ_(*a*_*t*_|*s*_*t*_)), which is the gradient of the policy function concerning the logarithm of the policy parameters. The second term in the gradient estimator is (∇_*a*_*t*__α*log*(π_ϕ_(*a*_*t*_|*s*_*t*_)) − ∇_*a*_*t*__*Q*(*s*_*t*_, *a*_*t*_))∇_ϕ_*f*_ϕ_(ϵ_*t*_; *s*_*t*_), which relates to the policy function The gradient of the logarithm to the action, the gradient of the action-value function to the action, and the gradient of the feature extractor to the policy parameters.

### 3.3 Partially Observable Markov Decision Processes (POMDPs)

Partially Observable Markov Decision Processes (POMDPs) (Tamar et al., 2016) is a broader framework than Markov Decision Processes (MDPs) for addressing planning challenges where an agent lacks complete knowledge of the environment's state. POMDPs represent decision-making scenarios wherein the agent's information about the environment is incomplete. A POMDP is mathematically defined as a tuple of six elements < *S, A, Z, P, O, R* >, where *S* is the state space, *A* is the action space, *Z* is the observation space, *P* is the state transfer function, *O* is the observation function, and *R* is the reward function. The observation function *O* maps a state-action pair to a probability distribution representing the likelihood of observing a particular observation. The transition function, denoted as *P*, represents the conditional probability of the environment transitioning from state *s*_*t*_ to state *s*_*t*+1_ given that the Agent executes action *a*_*t*_. The reward function *R*, denoted as *r*(*s*_*t*_, *a*_*t*_), characterizes the instantaneous reward that the Agent obtains upon executing a particular action in a specific state.

In the context of a partially observed Markov Decision Process (MDP), the Agent lacks direct access to the state of the environment. Instead, it receives an observation (*O*) that is contingent upon the underlying state (*p*(*o*_*t*_|*s*_*t*_)). Consequently, the Agent must rely on its observations to deduce the latent state of the environment and subsequently determine its actions. The Agent lacks direct access to the underlying state of the Markov Decision Process (MDP). In contrast, the Agent perceives the state indirectly through its observations, and as a result, the observation space may exhibit noise or incompleteness. The optimal Agent requires access to the entire history of the Agent's observations and actions, denoted *h*_*t*_ = (*o*_1_, *a*_1_, *o*_2_, *a*_2_, ⋯  , *a*_*t*−1_, *o*_*t*_), which is preferable to the MDP, in which the current state is dependent on all preceding states and actions taken by the Agent. Nevertheless, it may prove impractical or inefficient for an Agent to retain and process the complete chronicle of observations and actions. Hence, the Agent must employ a memory-based methodology to retain pertinent knowledge from previous instances while disregarding extraneous information.

Recurrent Neural Networks (RNNs) that have been trained using the Back Propagation Through Time (BPTT) algorithm are widely utilized in memory-based control within partially seen domains. Recurrent neural networks (RNNs) can retain a concealed state that encapsulates pertinent information from preceding instances, hence transmitting current actions to the Agent. The Long Short-Term Memory (LSTM) model is a Recurrent Neural Network (RNN) type that is highly proficient in capturing and modeling long-term dependencies within datasets. This study uses the backpropagation technique to train the Transformer model to capture memory-based control within a partially seen domain effectively. This study showcases the efficacy of the Transformer model in facilitating the Agent's resolution of diverse physical control challenges, which necessitate varying degrees of memory utilization. These challenges encompass integrating noisy sensor data in the short term and preserving information across multiple processes in long-term memory tasks.

In accordance with Equation (1), the objective function *J* is modified to represent the trajectory the stochastic policy must maximize in order to characterize it. Hence, the objective function *J* represents the anticipated accumulation of discounted benefits achieved over an unlimited time horizon in Equation (12).


(12) $$\begin{array}{l} J=E_{\tau }\left[\overset{\infty }{\underset{t=1}{\sum }}\gamma^{t-1}r\left(s_{t},a_{t}\right)\right] \end{array}$$

Trajectories τ are obtained from the distribution of trajectories generated by the method π. The trajectory distribution *p*(*s*_1_)*p*(*o*_1_|*s*_1_)π(*a*_1_|*h*_1_)*p*(*s*_2_|*s*_1_, *a*_1_)*p*(*o*_2_|*s*_2_)π(*a*_2_|*h*_2_)... can be expressed as the multiplication of three components: the initial state distribution *p*(*s*_1_), the observation distribution *p*(*o*_*t*_|*s*_*t*_), and the conditional action distribution π(*a*_*t*_|*h*_*t*_) conditioned on the history *h*_*t*_. The history *h*_*t*_ is an adequate summary of prior observations and actions up to time *t* − 1. The trajectory distribution refers to the distribution encompassing all conceivable trajectories τ = (*s*_1_, *o*_1_, *a*_1_, *s*_2_, *o*_2_, *a*_2_, ...) that can be produced by implementing the policy π based on the probability distribution. The objective function, denoted as *J*, quantifies the anticipated total reward acquired by adhering to the policy π during an unlimited time horizon. In this context, the reward is subject to discounting at each timestep by a factor of γ. In the context of a deterministic policy, the conventional policy function denoted as π is substituted with a deterministic function denoted as μ. This function directly maps the state *S* to the action *A*. Furthermore, in the conditional action distribution π(*a*_1_|*h*_1_), the history of action replacements replaces denoted as *h*_*t*_. This history is obtained by applying the deterministic policy function μ.

In the context of a fully observable Markov Decision Process (MDP), the Agent possesses knowledge of the current state *s*, and the action value function *Q*_π_ is established as the anticipated future discounted reward when the Agent takes an action in state *s*_*t*_ and after that adheres to policy π. In situations where observations are incomplete, the intelligence lacks access to the true state *s*, and the construction of the action-value function *Q*_π_ relies on the variable *h*. The variable *h* denotes the internal state or memory of the intelligence system, which undergoes updates at each timestep by the present observations and preceding internal states. The function *Q*_π_ is employed to assess the efficacy of the policy π, which serves as a mapping between states to actions. The primary objective of the algorithm is to identify the policy that will yield the highest possible predicted future discount reward. The algorithm utilizes the function *Q*_π_(*h*_*t*_, *a*_*t*_) to address control problems that involve incomplete observation, requiring the Agent to depend on its internal state for decision-making. This methodology enables the Agent to effectively incorporate data from imprecise sensors over time and preserve information at various temporal intervals, a crucial requirement for addressing distinct physical control challenges.

The *Q*-value function for a given policy π in a partially observed control situation is defined by Equation (13). The *Q*-value function quantifies the anticipated total reward that an Agent can obtain by adhering to the policy π in the context of a specific state-action pair (*h*_*t*_, *a*_*t*_).


(13) $$\begin{array}{l} Q_{\pi }\left(h_{t},a_{t}\right)=E_{s_{t}|h_{t}}\left[r_{t}\left(s_{t},a_{t}\right)\right]+E_{\tau_{>t}|h_{t},a_{t}}\left[\overset{\infty }{\underset{i=1}{\sum }}\gamma^{i}r\left(s_{t+i},a_{t+i}\right)\right] \end{array}$$

Equation (13) is comprised of two components: the instantaneous reward *r*_*t*_ acquired by executing an action in state *s*_*t*_, and the anticipated future reward acquired by adhering to the policy π for actions initiated from the subsequent state *s*_*t*+1_ and *a*_*t*+1_. The discounting of future benefits is denoted by the variable γ, which represents the inclination of the intelligence toward immediate rewards in comparison to delayed rewards. The future reward is determined by the complete sequence of states, observations, and actions, denoted as τ_>*t*_ = (*s*_*t*+1_, *o*_*t*+1_, *a*_*t*+1_, …), following the current state-action pair (*h*_*t*_, *a*_*t*_). The computation of this reward involves two expectations, which are conditioned on the probabilities *p*(*s*_*t*_|*h*_*t*_) and *p*(τ_>*t*_|*h*_*t*_, *a*_*t*_), respectively. These probabilities are evaluated based on the trajectory distribution the policy π induces. The trajectory distribution refers to the probability distribution, including all potential paths that can be pursued by an intelligent agent, based on the current state-action pair (*h*_*t*_, *a*_*t*_), while adhering to the on-policy π. The first expectation calculates the anticipated immediate reward that the Intelligent Agent can acquire by executing an action in state *s*_*t*_, considering the present belief state *h*_*t*_. The second expectation calculates the anticipated future reward that the Agent can achieve by adhering to the policy π from the subsequent state *s*_*t*+1_ and action *a*_*t*+1_, considering the present belief state *h*_*t*_ and action *a*_*t*+1_. The belief states, denoted as *h*_*t*_, serve as a comprehensive statistic that encapsulates all pertinent information on the intelligent Agent's previous observations and actions. These belief states are utilized to compute the trajectory distribution and the *Q* value function.

### 3.4 Transformer

Vaswani et al. (2017) and Parmar et al. (2018) first proposed Transformer in a research paper. The architectural design of the Transformer model is characterized by the utilization of stacked self-attention layers, which are interconnected via residual connections. In the context of self-attention (Choromanski et al., 2020; Wang et al., 2020) layers, it is observed that each layer gets a set of n embeddings denoted as ${\left\{x_{i}\right\}}_{i=1}^{n}$, where each embedding corresponds to a distinct input token. The self-attention layer subsequently generates *n* embeddings ${\left\{z_{i}\right\}}_{i=1}^{n}$, which maintain the original input dimension. Each token at index *i* is associated with a key *k*_*i*_, a query *q*_*i*_, and a value *v*_*i*_ through a linear transformation. The key *k*_*i*_ is essential for extracting pertinent information from the input sequence. Conversely, the query *q*_*i*_ calculates the attention scores between the key *k*_*i*_ and other keys. The value *v*_*i*_ is employed in calculating a weighted sum of attention scores, which is subsequently utilized in generating the output embedding ${\left\{z_{i}\right\}}_{i=1}^{n}$. Utilizing self-attention enables the model to selectively attend to various segments of the input sequence, which is highly advantageous in capturing distant relationships within the data. The utilization of residual connectivity addresses the issue of diminishing gradients and facilitates the efficient training of deeper architectures.

The self-attention layer is a crucial component of the Transformer architecture for sequence modeling tasks such as language translation and sentiment analysis. In the context of the Decision Transformer, the Self-Attention Layer is used to compute the optimal action based on the input sequence of states and actions in Equation (14).


(14) $$\begin{array}{l} z_{i}=\overset{n}{\underset{j=1}{\sum }}softmax{\left({\left\{<q_{i},k_{j^{\prime }}>\right\}}_{j^{\prime }=1}^{n}\right)}_{j}\cdot v_{j} \end{array}$$

The self-attention (Yoo et al., 2015) layer operates by calculating a weighted summation of values *v*_*j*_, with the weights determined by the normalized dot product between the query *q*_*i*_ and the remaining keys *k*_*j*_. The query *q*_*i*_ represents the present state or action, whereas the key *k*_*j*_ represents a previous state or action. Higher values indicate more significant similarity between the query *q*_*i*_ and each key *k*_*j*_, as measured by the dot product. The dot product is subsequently normalized using the softmax function, which guarantees that the weights are normalized to a sum of 1 and accurately represent the probability distribution over the keys. Subsequently, the obtained weights are employed to provide a weight to the value *v*_*j*_, which signifies a characteristic or representation of a previous condition or action. The *i*th output of the self-attention layer is determined by the weighted sum of the *v*_*j*_ values. This output calculates the optimal action for the current state or action. In general, incorporating a self-attention layer in the Decision Transformer model enables the model to effectively capture interdependencies among the pieces of the input sequence, hence facilitating the generation of optimal actions driven by the expected return or reward.

The VIT (Dosovitskiy et al., 2020) architecture is a variant of the Transformer architecture, a prevalent framework employed in several natural language processing applications, including language modeling and machine translation. The Transformer architecture comprises a neural network with multiple layers of self-attention and feed-forward mechanisms. This design enables the model to capture long-range dependencies within the input sequence. Utilizing the self-attention mechanism enables the model to choose to attend to various segments within the input sequence and calculate a weighted summation of the input embeddings, considering their interrelationships. The VIT architecture modifies the Transformer model by incorporating a causal self-attention mask. This mask constrains the attention mechanism to attend solely to preceding tokens in the sequence throughout the training and generation processes. The VIT model can construct autoregressive sequences, wherein each token is formed by considering the preceding token. The similarity of query vectors and key vectors in the self-attention mechanism enables the model to implicitly form state-return associations, where similar states are associated with similar returns. The computation of attention weights, which determine the relative relevance of each input token for the output, is achieved by taking the dot product between the query vectors and key vectors. Using the VIT architecture within the Decision Transformer framework enables the model to generate future actions that generate the desired returns by adjusting the autoregressive model to the desired returns, past states, and actions.

## 4 Deep reinforcement learning navigation via decision transformer

This study presents the DRLNDT approach, which combines decision transformer with deep reinforcement learning to achieve motion navigation in autonomous vehicles. The objective is to successfully guide the vehicle from its starting point to its final destination. This approach enables processing high-dimensional observations by utilizing pixel-level learning from raw, high-resolution photos captured by the autonomous vehicle.

### 4.1 Deep reinforcement learning navigation via decision transformer backgrounds

In the context of our autonomous driving navigation task, which heavily relies on visual perception to understand the surrounding environment, it is necessary to consider the potential limitations of static photographs in conveying information about the speed of dynamic situations. The occlusion of objects can occur as a result of the inherent three-dimensional characteristics of the environment. In addition, most visual sensors have limited bandwidth, limiting the Agent's ability to perceive the environment accurately and influencing the self-driving car's navigational decision-making capabilities. The SAC algorithm is a fundamental deep reinforcement learning method employed in our research. A concise overview of its central ideas may be found in Section 3.2.

This paper examines an extension of SAC to Partial Markov Decision Processes for partially observed image data processing in autonomous navigation. The fundamental concept underlying the SAC method is iteratively adjusting the policy parameters in the direction of the gradient of the predicted reward for these parameters. In situations where observation is limited, the accurate estimation of the action-value function becomes unattainable. Consequently, the policy must be adjusted based on the observed condition and reward. The credibility of designating a state as a current observation is uncertain, thus necessitating the inference of the present state of the environment based on a chronological record of past observations up to the present moment. In order to tackle this issue, a Transformer model is employed to encode policy that retains information from previous observations and actions, which are subsequently trained using the backpropagation algorithm. The neural network serves as a function approximator to accommodate huge observation spaces, such as the pixel space acquired through integrating a camera into an autopilot system. The use of neural networks with convolutional measures in this method has proven effective for various perceptual processing tasks and for extending reinforcement learning to significant state space methods.

Using neural networks with convolutional measures has proven effective in this approach for various perceptual processing tasks and extending reinforcement learning to extensive state space methods. In our autonomous driving navigation task, however, we use an infinite space of pixels and high-quality images with a high degree of information, not only because more features can be extracted from high-quality images but also because we want our algorithms to apply to real-world environments. The high quality of images captured by the camera of a real car leads to a significant memory requirement during training. Consequently, setting greater values for the *buffer*_*size* and *batch*_*size* becomes impractical, adversely impacting the training results and efficiency. The variational autoencoder (VAE) is employed to extract latent vectors. The conversion of the image space to latent space is performed to establish a congruence between the latent vector space and the image space within the machine's cognitive framework. Consequently, using the latent space instead of the image space ensures the preservation of characteristics to the greatest extent possible while minimizing the memory footprint. The term is denoted as *S*_*latent*_.

Determining the optimal policy and action-value function is contingent upon the historical record of previously observed actions, represented as *h*_*t*_. In this study, we suggest a modification to the neural network framework that facilitates the acquisition of knowledge regarding the policy and action-value function. In this study, we suggest employing a Transformer network as an alternative to a feed-forward network. Preserving messages with history is an essential capability of the Transformer model, as it enables the resolution of partially observed situations.

The utilization of Transformer (Choromanski et al., 2020; Ding et al., 2020; Parisotto et al., 2020) enables the formulation of the policy and action value functions, represented as π(*h, a*) and *Q*(*h, a*) respectively, in terms of the observed action history *h*_*t*_. This approach facilitates the policy update process by incorporating the history of observed actions rather than solely relying on the current observation (*o*_*t*_). Additionally, it allows for utilizing the learned approximation (*o*_θ_) to address the challenges posed by the partially-observed control problem *Q*_θ_, thereby replacing *Q*_π_.

DRLNDT is off-policy, indicating that the policy being learned differs from the policy used to generate the data. Exploration is necessary for acquiring knowledge about the gradient of the *Q* function to actions. This approach necessitates that the Agent do behaviors that can be more optimal to acquire knowledge of the environment. However, exploration can be inefficient and unpredictable in practice. To address this issue, academics frequently employ experience replay to enhance data efficacy and stability. The process of experience replay entails the storage of experience trajectories in memory and the subsequent sampling from this memory throughout the learning phase. This approach enables the Agent to acquire knowledge from diverse encounters and has the potential to enhance the robustness of the learning process. In the case of DRLNDT, sampled memory trajectories are used to learn expectations through experience replay. In our memory, we store a tuple < *O*_*t*_, *A*_*t*_, *O*_*t*+1_, *R*_*t*_, *done* >. Here, *O*_*t*_ represents a succession of observations labeled as *o*_*t*−*n*_, *o*_*t*−*n*−1_, ..., *o*_*t*_. This sequence is continuous and differs from the conventional representation of *O*_*t*_. In our case, *O*_*t*_ encompasses past and present observations. The set *A*_*t*_ is defined as the collection of *a*_*t*_ values that exclusively represent the action associated with the present state.

### 4.2 Baseline architecture

This study conducts a comparative analysis of two methodologies for training Agents using reinforcement learning to achieve autonomous navigation from the starting point to the final destination in the context of autonomous driving. The first methodology discussed in the paper is called deep reinforcement learning navigation via decision transformer (DRLNDT). The second methodology employed in this study involves utilizing a recurrent neural network (RNN) to encode time series data. The second strategy is designated as the baseline approach, and our study primarily focuses on conducting controlled experiments using this approach.

**Figure 1:**
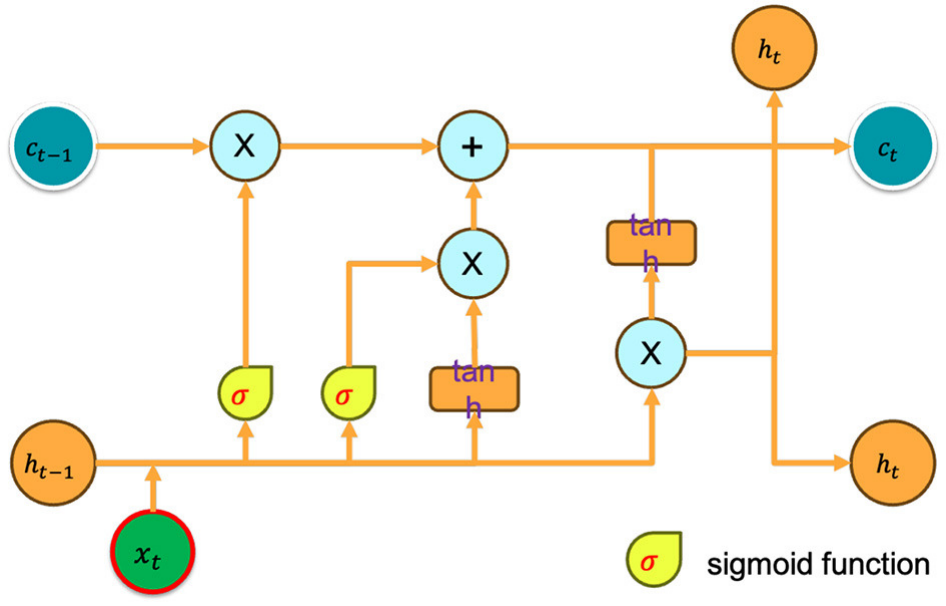
Long Short-Term Memory (LSTM) layer.

Deep reinforcement learning (DRL) has demonstrated efficacy in contexts with complete observability, although its performance has been suboptimal in environments with partial observability. In 2015, Hausknecht and Stone (Hausknecht and Stone, 2015) introduced a system known as Deep Recurrent *Q*-Learning (DRQN) as a potential solution to tackle the issue above. The proposed modification involves substituting the initial fully-connected layer after the convolutional layer in a conventional Deep Q-Network (DQN) architecture with a Long Short-Term Memory (LSTM) (Yu et al., 2019) layer as shown in Figure 1. In contrast to Deep Q-Networks (DQNs), which rely on fixed-length histories, the Deep Recurrent Q-Network (DRQN) has a recurrent structure that allows for the integration of arbitrarily lengthy histories, enhancing the accuracy of current state estimation. DRQN estimation function *Q*(*o*_*t*_, *h*_*t*_ − 1|*theta*) rather than *Q*(*s*_*t*_, *a*_*t*_|*theta*), where *theta* represents the network parameters, *h*_*t*_ − 1 represents the output of the *LSTM* layer in the previous step, and *h*_*t*_ = *LSTM*(*h*_*t*_ − 1, *o*_*t*_). The performance of *DRQN* on the standard *MDP* problem is comparable to that of *DQN*, while *DRQN* outperforms *DQN* in partially observable domains. The algorithm is enhanced by integrating the Recurrent Neural Network (RNN) with the Soft Actor-Critic (SAC) framework. This is achieved by utilizing the function *Q*(*o*_*t*_, *h*_*t*−1_, *a*|θ) instead of *Q*(*s*_*t*_, *a*_*t*_) to estimate the *Q*-function, and employing π(*a*_*t*_|*h*_*t*−1_, *o*_*t*_, ϕ) instead of π(*a*_*t*_|*s*_*t*_) to estimate the policy function. This methodology is defined as baseline algorithm.

### 4.3 DRLNDT architecture

Let us compare our approach to the baseline method. The Transformer layer is included in our approach to incorporate the historical context, enhancing the accuracy of forecasting the present state. The estimation of the *Q* function and policy function is performed using the current action. The structural architecture of DRLNDT exhibits notable similarities to that of DRQN. This resemblance arises from utilizing the Transformer layer to integrate historical information, adopting time as a positional reference, and incorporating the Attention mechanism. These features enable the integration of past and future information at a specific temporal point, a capability not attainable in RNN networks.

In conjunction with the principle of maximum entropy, neural networks are employed to approximate the value and policy functions to acquire knowledge about the optimal policy. Initially, we present the value network concept, wherein the inputs consist of states and actions. Since the state is unknown and we can acquire observations from the surrounding environment, we must determine the actual state from the observations. Observations refer to the data obtained by directly utilizing sensors embedded within the autopilot system. On the other hand, the state represents what has been learned derived from past observations. Due to numerous sensors, the state obtained by the autopilot from the surrounding environment in our self-driving car exhibits multimodality. The camera is a primary sensor utilized by autonomous vehicles. Autonomous driving aims to enable vehicles to navigate their surroundings by utilizing camera-based perception systems, thereby emulating human-like image-based driving capabilities. Images are employed as the principal state space for multimodal states. The remaining modal states encompass state vectors that include velocity, acceleration, position, and distance relative to the final position of the autonomous vehicle. Hence, combining the image space and the state vectors constitutes the multimodal state space. Nevertheless, we have identified an additional issue of using the image as a state space. The utilization of a replay buffer necessitates the storage of state information in memory. However, the substantial memory requirements associated with high-quality images, specifically those with dimensions of 640 * 640, restrict the ability to increase the *buffer*_*size* beyond a certain threshold. The *batch*_*size* parameter is employed during the training process in order to enhance the efficiency of training. Furthermore, converting high-quality images into tensors results in a more significant memory allocation for the image matrix, thereby imposing greater demands on the GPU hardware due to the increased GPU memory consumption.

Consequently, the utilization of high-quality images does not yield increased efficacy in the process of training. In our experimental evaluations, it was observed that the utilization of a computing system that only marginally satisfies the hardware prerequisites results in a notable decrease in the training efficiency of the model. As an illustration, the computational efficiency of a 64 * 64 image surpasses that of a 640 * 640 image by a factor of three, a performance level that does not meet our requirements. Nevertheless, we must continue to explore the utilization of high-quality images as a means of perceiving and comprehending the surrounding environment. The utilization of high-quality images enables the machine to capture finer details that may not be discernible to the machine, thereby enhancing the intelligence's ability to perceive the environment more realistically, as compared with low-quality images. To solve the problem of memory consumption by high-quality images, we use VAE to obtain latent states from high-quality images. After VAE processing, the latent state is considered by the machine to be consistent with the high-quality image. As a result, we use VAE to compress high-quality images with minimal image loss, thereby decreasing memory consumption. Therefore, we ingeniously deduce the latent state and trick the machine into believing that the latent state is consistent with the image. The retention of image information is maximized while minimizing memory consumption. Consequently, our multimodal state space consists of two states, latent state and vector state, which can be obtained easily from the sensor.

**Figure 2:**
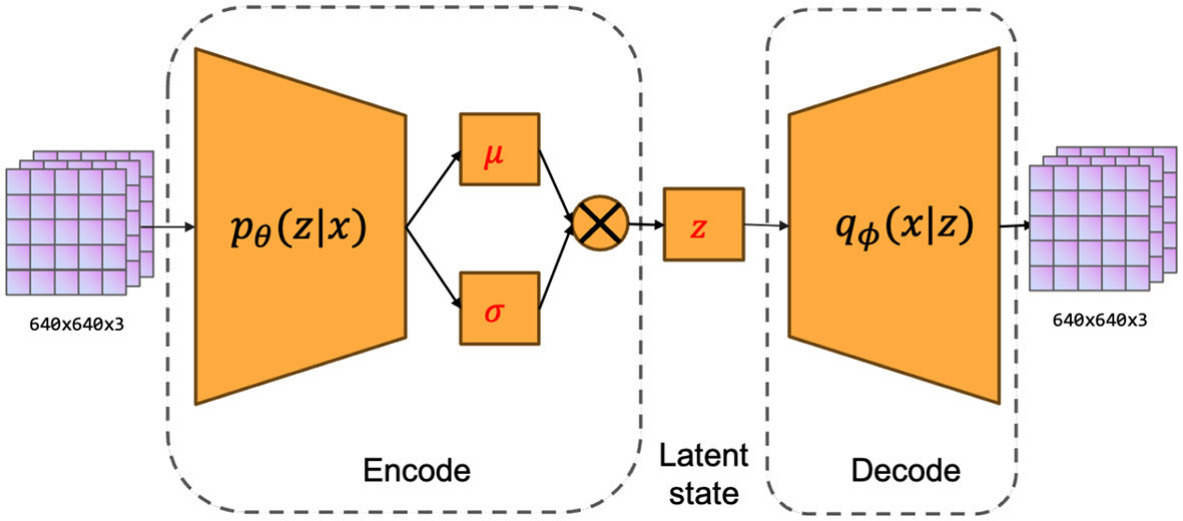
The network structure of VAE includes two parts: encoder and decoder.

The fundamental concept underlying VAE is to map the input data into a low-dimensional latent space and reconstruct the vectors of the latent space using a decoder into samples similar to the original data. The variational autoencoder (VAE) is comprised of two main components, namely an encoder and a decoder. The process of encoding involves mapping the input data to the statistical measures of the mean and variance in the latent space. On the other hand, the decoding process generates novel samples by utilizing vectors that are randomly sampled from the latent space. The network architecture of the variational autoencoder (VAE) is depicted in Figure 2. The system can be primarily categorized into two components, namely, the encoder and the decoder. The encoder component is tasked with converting the input image into a latent state representation with reduced dimensions. The dimensions of the input image are 640 pixels by 640 pixels, and it consists of three color channels (red, green, and blue). The encoder is composed of a sequence of convolutional layers and an activation function that progressively decreases the spatial dimensions of the input image and captures valuable features. The activation function employed in this process is LeakyReLU. The result of the encoder is fed into the Flatten layer in order to obtain a vector with one dimension. The dimension of the latent vector is a determining factor for its overall dimensionality. In our research paper, we decided to use a latent vector dimension of 256 in order to strike a balance between expressive capacity and dimensional efficiency. The decoder component transforms the latent vector into the image space, resulting in a reconstructed image that maintains the exact dimensions as the original input image. The ultimate layer of the decoder employs a hyperbolic tangent activation function (Tanh) to guarantee that the resultant pixel values fall within the range of -1 and 1, aligning with the input image's range. Consequently, the self-driving car engages with the environment to acquire high-quality images, which are subsequently processed by the variational autoencoder (VAE). These processed images are then extracted as latent vectors, referred to as latent states within our algorithm.

**Figure 3:**
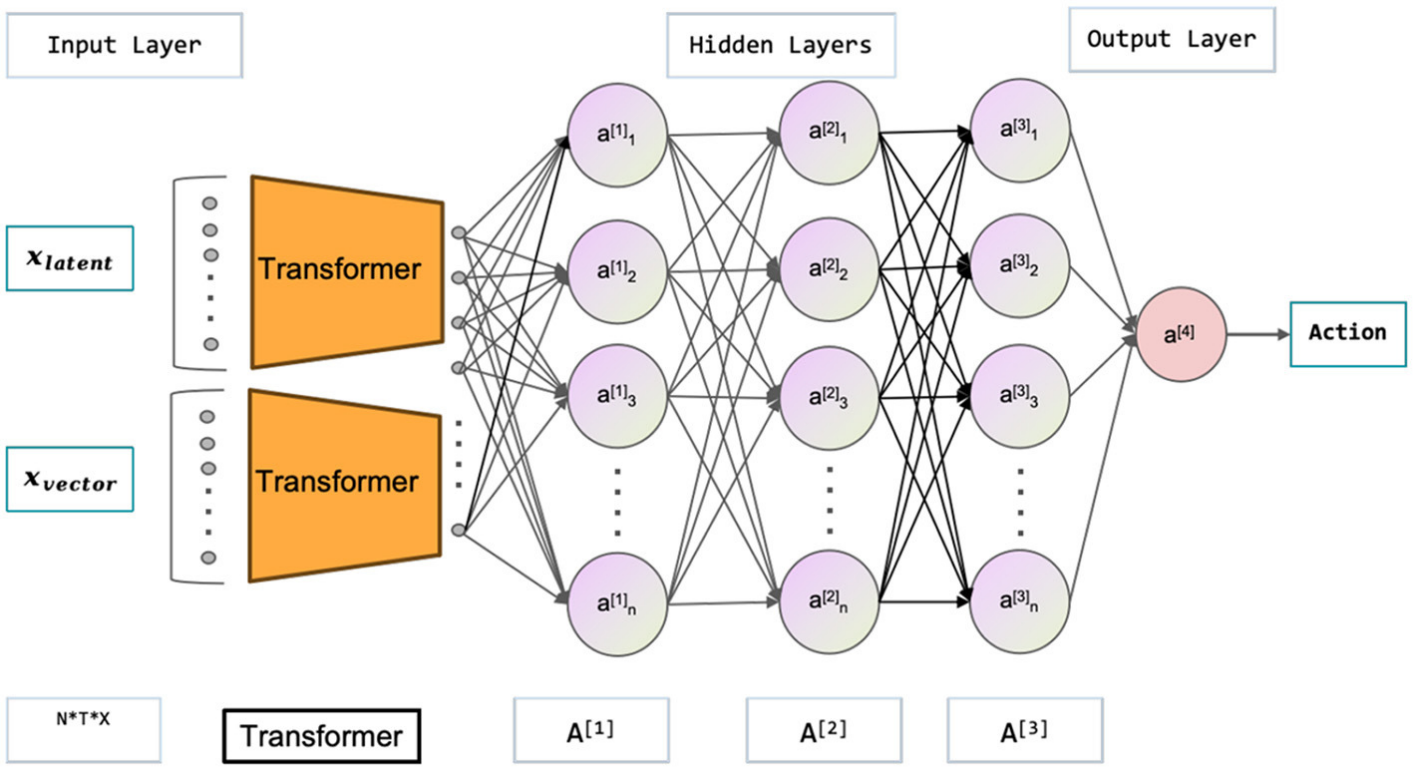
The figure shown represents the Actor-Network in the context of the Soft Actor-Critic (SAC) paradigm, which is used to learn policies. The inputs are the latent states *x*_*latent*_ and vector states *x*_*vector*_, while the outputs are the actions. The latent states are high-quality images obtained through variational autoencoder (VAE) processing.

**Figure 4:**
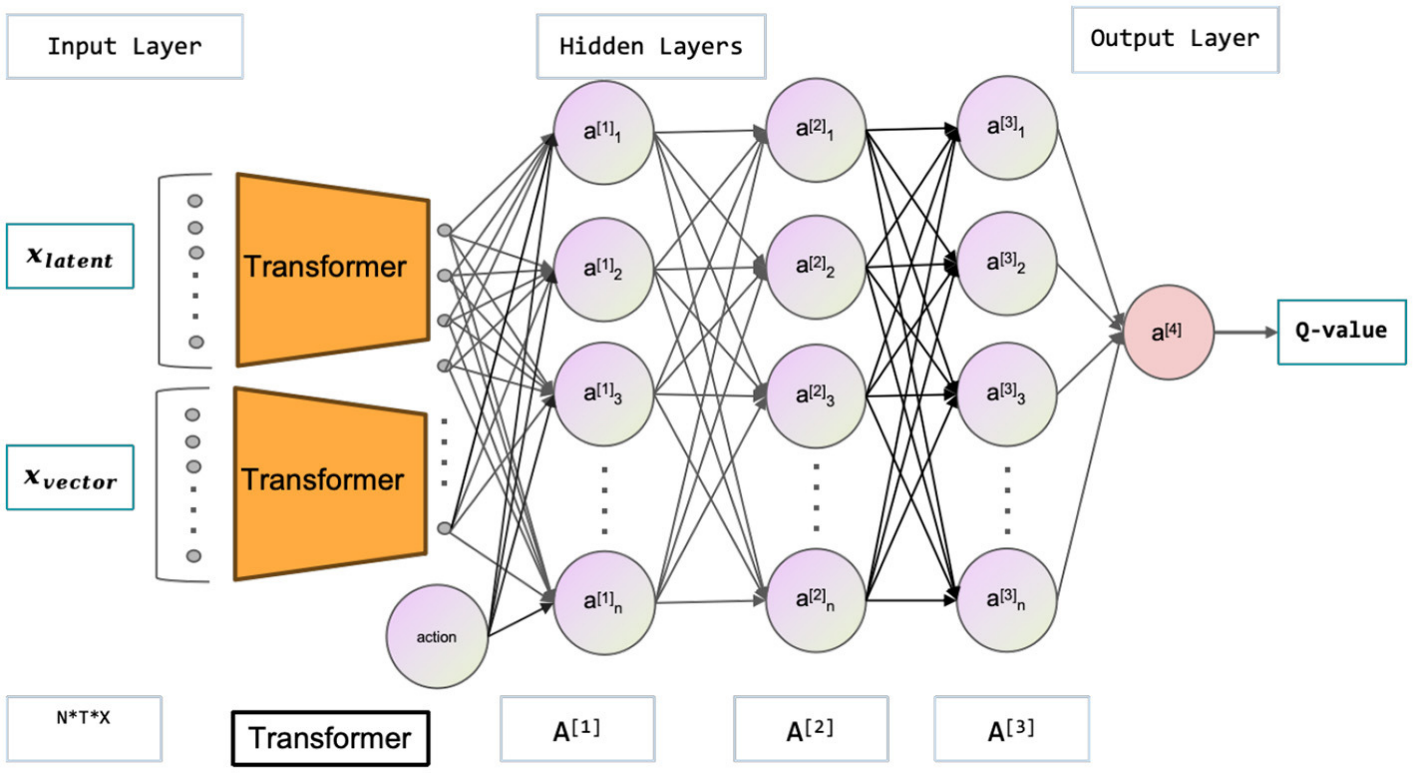
This figure depicts the Critic network used in the Soft Actor-Critic (SAC) model. Its main purpose is to evaluate the quality of policies. The inputs are the latent states *x*_*latent*_ and vector states *x*_*vector*_, while the outputs correspond to *Q*-values. The latent states refer to high-quality images processed through a variational autoencoder (VAE).

The multimodal state space is comprised of the latent and vector states, which, in conjunction with the current action, are utilized to estimate the current value function and policy function. Neural networks are employed to approximate both the value function and policy function, as depicted in the Figures 3, 4. illustrating the network architecture for these functions. The multimodal state space is temporally superimposed, encompassing historical data pertaining to the state space. Consequently, the most recent state space is redefined as the historical state space, thereby distinguishing it from the conventional state space. The concept of historical state space refers to a sequential arrangement of states over time, encompassing both past-to-present time observations and a partial trajectory of the past. The time series can be characterized by the historical latent states, which are derived from the image state space. Furthermore, by analyzing the state space of the history vector, one can derive the velocity, the positional relationship, and other pertinent information. In the context of multimodal state space, it is possible to establish a correspondence between the information pertaining to velocity, position, and other relevant variables obtained from an image and the corresponding information in the vector space. This correspondence enables the extraction of features such as velocity from the image. Extracting features related to velocity and position from a single state becomes challenging, particularly in the presence of occlusion. The algorithmic framework has been transformed from a Markov Decision Process (MDP) to a Partially Observable Markov Decision Process (POMDP). Hence, it is necessary to derive the actual state space from the partially observed historical state space. Experimental proof supports the notion that obtaining the optimal policy is more feasible using the historical state space.

**Figure 5:**
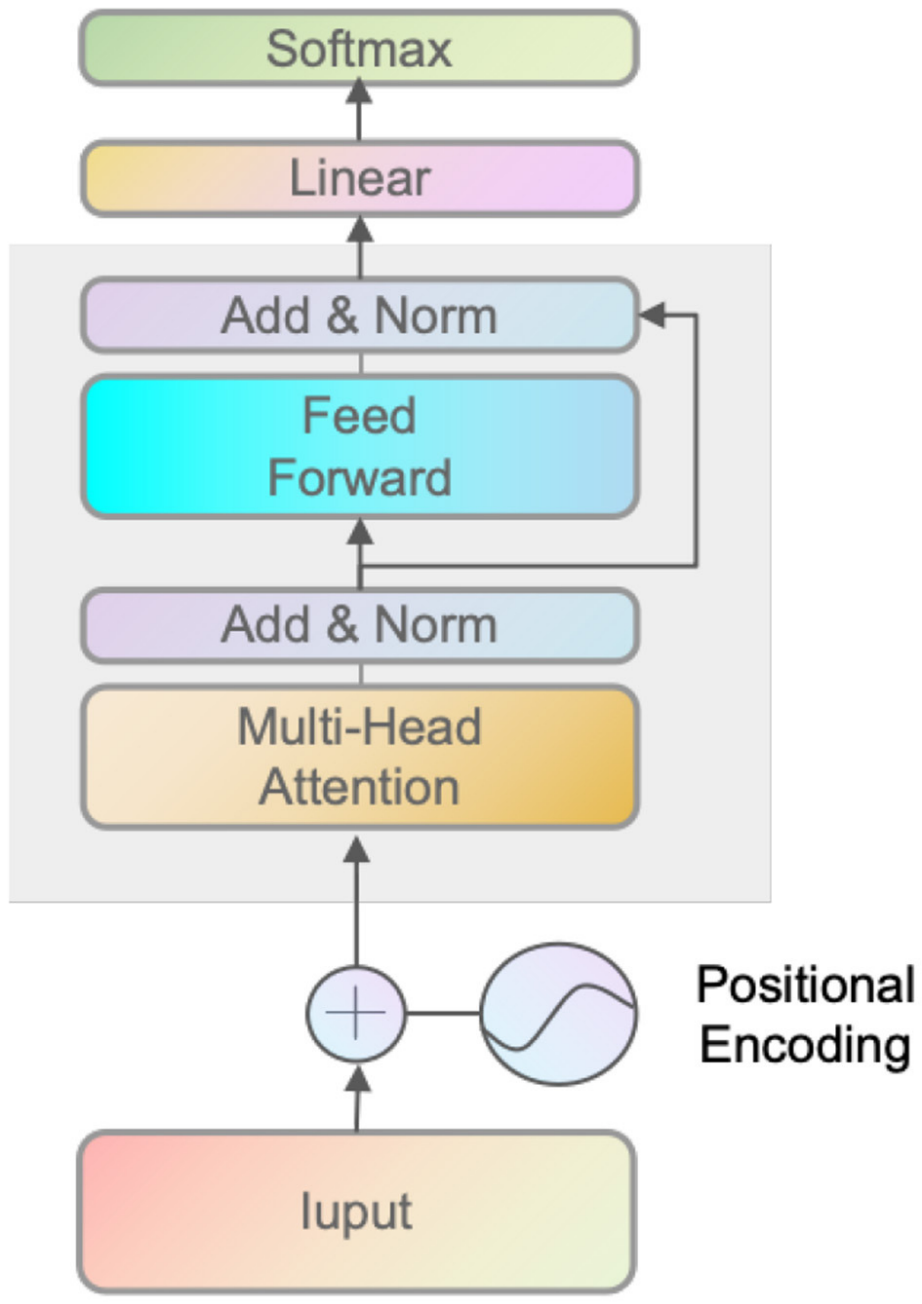
The figure illustrates the Transformer network's coding structure. The input is a continuous time series of latent vectors *x*_*latent*_ and vector states *x*_*vector*_ defined as historical states.

The process of extracting the current state from a given historical context is a topic of interest. The initial step in the structure of the value function and policy function involves extracting the current state from the historical data. In the implemented algorithm, a Transformer model is employed to extract the current state from the historical data. Subsequently, a multilayer neural network is employed to approximate both the value function and policy function. The division of the value function and policy function is comprised of two components. The first component is referred to as the transformer module, which is illustrated in Figure 5. The second component is a multilayer neural network, where the activation function employed is LeakyReLU. The final layer of the policy function incorporates the Tanh activation function in order to confine the output within the range of −1 and 1, aligning with the permissible values for the output action. The output action is composed of two dimensions. *Action 1* involves a range of values from 0 to −1 for executing a left turn and a range of values from 0 to 1 for executing a right turn. *Action 2* involves adjusting the brake input from a value of 0 to −1 and the throttle input from a value of 0 to 1. The results of the final experiments indicate that our proposed method exhibits superior performance compared to LSTM's time series prediction in addressing incomplete observations. Additionally, the learned autopilot policy demonstrates better performance than the baseline method.

## 5 Experiment

To ascertain the efficacy of our approach in acquiring more optimal policies, we conduct a validation of our algorithm within the CARLA (Dosovitskiy et al., 2017) simulation environment. The empirical findings indicate that the DRLNDT algorithm exhibits superior learning capabilities in deriving optimal policies from historical data, surpassing both the baseline method and other policy methods that lack access to such data.

### 5.1 Simulation environment

Given the inherent characteristics of reinforcement learning algorithms, it is imperative for self-driving vehicles designed to operate autonomously to engage in continuous interaction with their surrounding environment. Due to the high cost and lack of security associated with fundamental interactions, we cannot train the algorithms in a natural environment using actual vehicles for learning. The CARLA (Dosovitskiy et al., 2017) simulation environment, which is already established and known for its realistic qualities, is utilized for both training and testing the algorithm. CARLA is an open-source simulator designed for autonomous driving systems, utilizing the Unreal Engine 4 platform for its development. The application offers a practical and adaptable three-dimensional setting wherein researchers and developers can assess and optimize their algorithms, eliminating the necessity for physical vehicles. The CARLA simulation environment offers users high-fidelity, authentic urban settings that encompass dynamic traffic, pedestrians, diverse weather conditions, and a range of road configurations.

Furthermore, it provides support for a diverse range of sensor models, encompassing LIDAR, millimeter wave radar, cameras, and other such technologies. The CARLA platform offers a map editor tool that facilitates the creation and modification of diverse road networks, buildings, and other components within a given scene. CARLA additionally offers a comprehensive range of application programming interfaces (APIs) and tools, facilitating the expeditious development and evaluation of autonomous driving algorithms by researchers and developers. Hence, the CARLA simulation environment is employed in order to acquire information and evaluate the DRLNDT algorithm, thereby confirming the superiority of our approach over the baseline method and its capability to acquire a more optimal policy.

### 5.2 Simulation environment configuration

**Figure 6:**
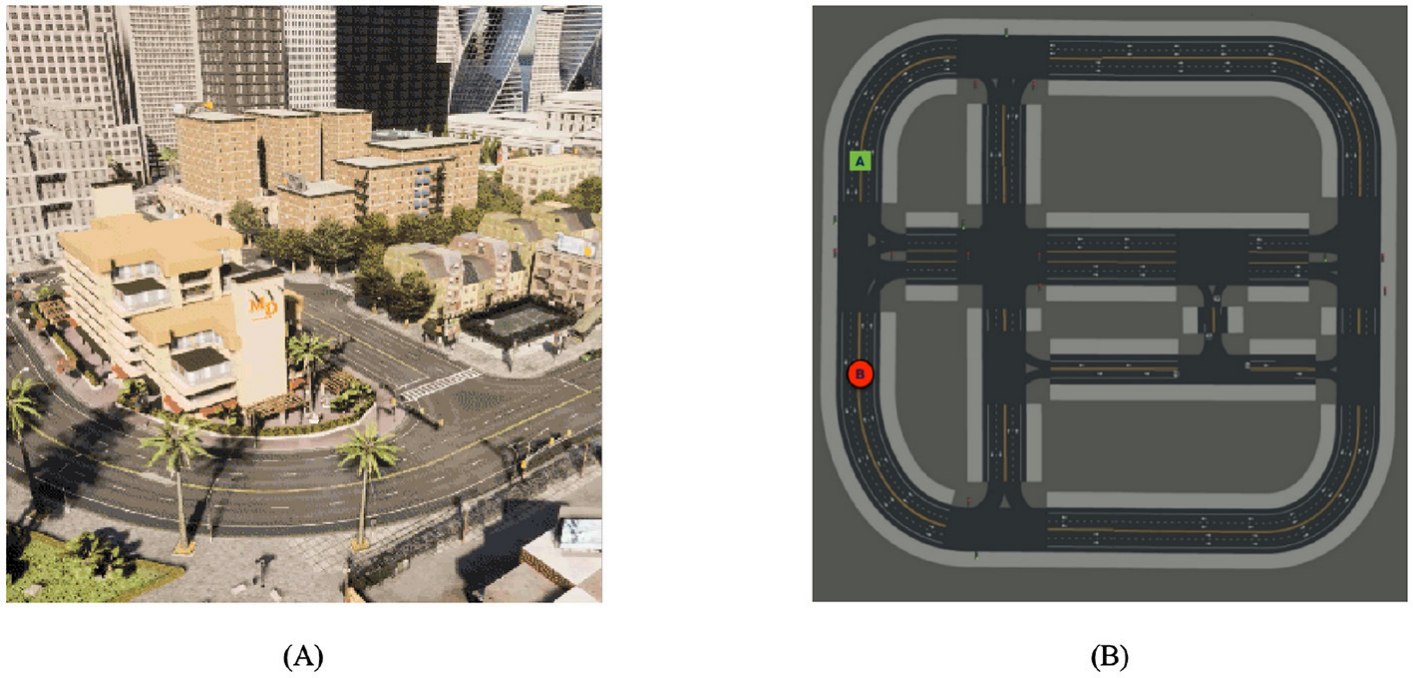
**(A)** Shows the simulation effect of the Town10 map in the CARLA simulation environment, portraying various structures. On the other hand, **(B)** presents a highly precise map of Town10HD in Carla. This figure is annotated to provide information on the initial and end coordinates.

The selected operating environment for CARLA is Ubuntu 20.04, equipped with a 64GB RAM and an NVIDIA 3090 GPU. This configuration fulfills the necessary specifications for both CARLA and our algorithms. Our research team has selected Town10HD in CARLA as the designated simulation environment for our study. This particular environment encompasses a comprehensive representation of a town, including a diverse range of buildings and road infrastructure. The complexity of the town environment is depicted in the high precision map of Town10HD, as illustrated in the Figure 6. Within the simulated environment, we established a standardized condition of clear, sunny weather during daylight hours. This condition deliberately excludes the presence of fog or rain, ensuring that the environmental factors remain unobscured. Vehicles are allowed to drive from the starting point to the finishing point without having to follow established traffic rules. The autonomous vehicle has the capability to navigate from its starting point to its destination along any possible path. This approach subsequently decreases the regulatory limitations for the autonomous driving system, so enabling it to possess greater adaptability and flexibility. The high-precision map is annotated with the starting and ending coordinates. The accomplishment of this assignment is readily attainable by current conventional approaches. Nevertheless, the development of a self-learning-based intelligent body autonomous driving system poses significant challenges. The state space inside urban areas exhibits a high degree of complexity and contains an unlimited dimension. The objective of our endeavor is to enable an autonomous agent to obtain the optimal policy and imitate human-like driving behavior only based on camera images and readily available state vectors in an autonomous vehicle. Our study primarily centers around the acquisition of driving skills through human-like intelligence, which is crucial for the development of reinforcement learning-based autonomous navigation systems in the context of autonomous driving.

**Figure 7:**
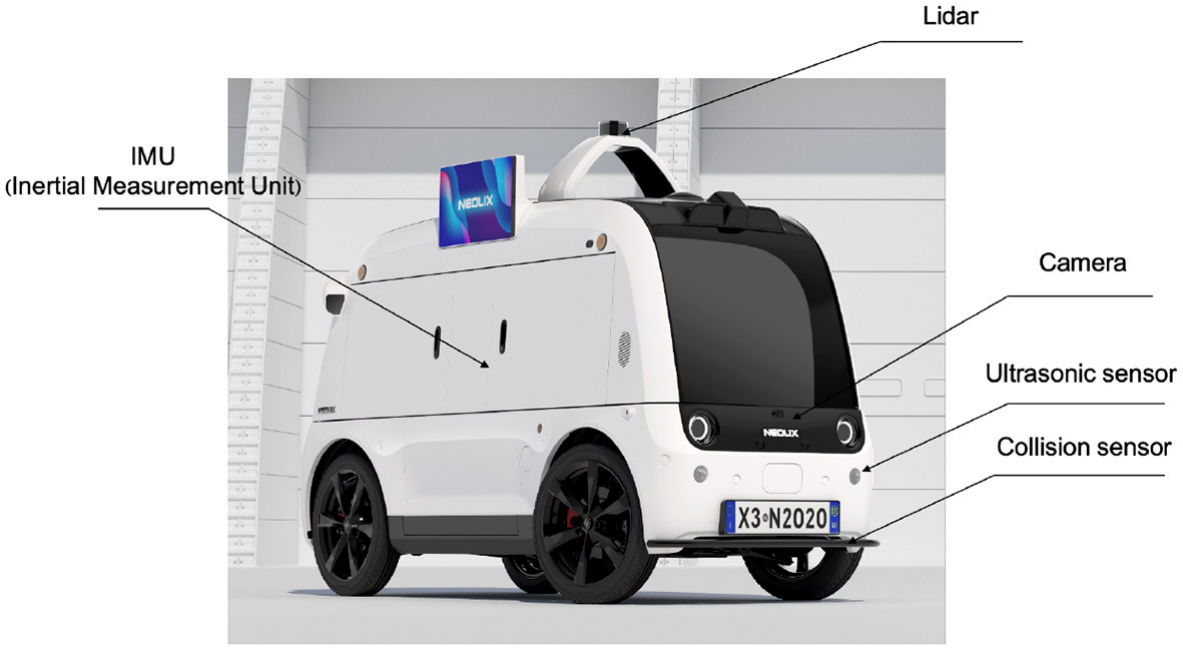
The figure illustrates the arrangement of sensors on a practical vehicle. The sensors utilized in this system primarily consist of a camera, ultrasonic sensor, speed encoder, Inertial Measurement Unit (IMU), and collision sensor.

In CARLA, we configure the simulated vehicles in accordance with the research requirements. As the foundation for the simulated vehicle, we utilized a Tesla model3. A camera with a resolution of 640*640*3 is installed on the roof in a position directly facing the car, with the purpose of capturing visual data from the area directly in front of the vehicle. Similar to human driving, an autonomous system operates the vehicle by maintaining focus on the road ahead. Therefore, the camera on the vehicle is able to detect the surrounding environment and acquire the image state of the Agent. Other vector states of the Agent, such as velocity and position, can be acquired directly via the vehicle API in CARLA, so additional sensors are not required. Therefore, our algorithm in CARLA simulates autonomous navigation without requiring information from high-precision maps. As a result, our algorithm does not rely on high-precision maps and relies mainly on the camera's acquired images to understand the driving policy, just as humans do. Applied to a physical vehicle, as shown in the Figure 7, it may be necessary to install sensors such as GPS, IMU, and encoder to acquire the vehicle's position and speed information without relying on a high-precision map. Our algorithm reduces the need for high-precision maps, which further reduces the operating costs of the autonomous driving system, and investigates the intelligence of autonomous driving navigation algorithms.

### 5.3 Training

Now, it is necessary to train our method in the CARLA environment in order to learn the optimal policy for navigating from the initial position to the final position by simulating interactions with autonomous vehicles in the environment. First, we must train the VAE model so that it can extract the latent states from the image states. Thus, we guarantee the accuracy of the image data while minimizing memory usage. Then, we devise the reward function, which, instead of restricting the autonomous vehicle's driving behavior, guides it to drive in accordance with human preferences. In conclusion, we operate the autonomous vehicle as an Agent in driving that learns the optimal policy through continuous interaction with its environment.

#### 5.3.1 Variational autoencoder training

**Figure 8:**
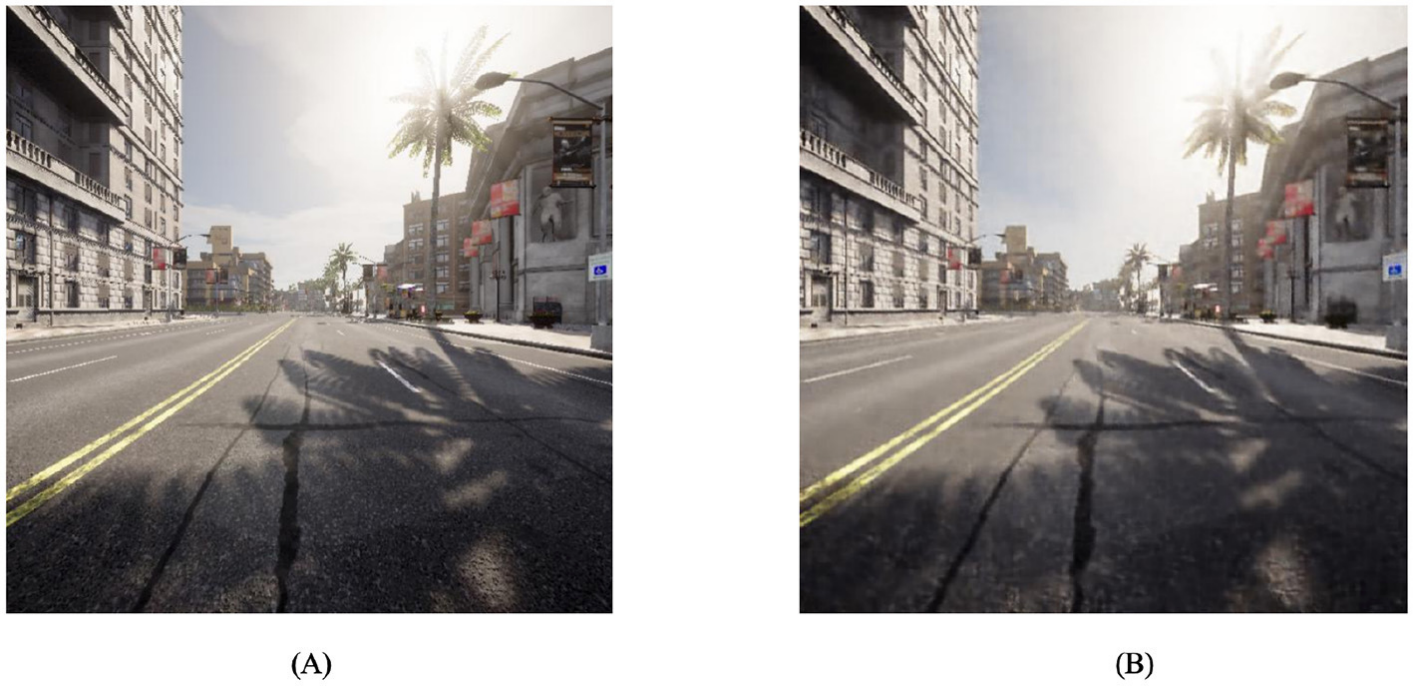
The variational autoencoder (VAE) experimental results are presented in the figure. **(A)** Shows the input image, which has a resolution of 640*640 and is of excellent quality. On the other hand, **(B)** illustrates the output image of good quality with the same resolution of 640*640.

Initially, it is necessary to gather the requisite dataset for training the variational autoencoder (VAE). The primary purpose of our VAE model is to analyze the picture data captured by the camera integrated into the autopilot system in order to extract the latent state. Hence, it is necessary to utilize a dataset consisting of photographs pertaining to the environmental makeup of CARLA towns and cities for the purpose of training the VAE. This approach facilitates enhanced data processing capabilities. There are two distinct methodologies for obtaining data inside the dataset utilized for training VAE. The first approach involves configuring the autonomous car with a random strategy, enabling it to navigate aimlessly from its starting place. This approach allows the vehicle to gather information about the town's unfamiliar environment. The second approach involves employing an optimal policy to gather data, hence mitigating the risk of data imbalance and insufficient destination data. Given our knowledge of the starting and ending positions, the best strategy can be readily comprehended by human beings, allowing for straightforward evaluation and implementation using conventional approaches. The acquired data is stored in an offline local storage. Subsequently, the data from both ways is combined to create a unified dataset. Random sampling is then employed from this dataset to train the VAE model. The size of the collected dataset was determined to be 32,768, while the *batch*_*size* was set to 16 in order to meet the training requirements. The training process was terminated after reaching a total of 100,000 training cycles. The mean squared error (MSE) is computed in order to satisfy our specified criteria. The experimental findings are depicted in Figure 8. The VAE produced from our training is utilized to extract the latent state, which aims to minimize memory usage while preserving the original features to the greatest extent possible.

#### 5.3.2 Reward design

Autonomous navigation algorithms for autonomous vehicles that are based on reinforcement learning diverge from traditional methods. This approach enables an Agent to autonomously acquire information by engaging with the environment and determining the optimal policy through the utilization of rewards obtained from the environment. Hence, this algorithmic technique diverges significantly from the conventional methodology, wherein predefined rules and logic are established to govern the behavior of autonomous vehicles in order to execute tasks. This method is mechanical and needs more intelligence. Nevertheless, our methodology entails directing the Agent to execute our objectives through the design of rewards rather than restricting it solely to the final result. This strategy enhances the Agent's level of unpredictability and intelligence. The system is capable of making varied decisions in response to diverse environmental conditions. It is also the reason why the optimal policies acquired using these algorithms possess the capacity to generate behavioral strategies that exceed those exhibited by human drivers. This cognitive approach can be employed to foster innovative and unconventional thinking beyond the conventional boundaries of human cognition.

The reward function was designed with the purpose of providing guidance to autonomous driving systems in order to successfully accomplish the task of navigating from the starting point to the ending position. The primary objective of the navigation challenge is for the autonomous vehicle to successfully arrive at the designated end location. Upon reaching the designated destination, the autonomous vehicle will be rewarded with a reward that is denoted as *r*_*success*_ = +1 for successfully completing its task. Simultaneously, upon the successful completion of the mission, the episode concludes, marking the finish of a training cycle. In the event of a collision involving the vehicle, the Agent will receive a negative reward denoted as *r*_*collision*_ = −3. This reward is implemented as a preventive measure to discourage the Agent from engaging in collisions. Simultaneously, the task is unsuccessful, resulting in the conclusion of the episode and the fulfillment of the training cycle. Subsequently, in order to guarantee the duration of the training episode, the training episode concludes when the duration of the episode surpasses the value of *n*. The Agent is subjected to a negative reward denoted as *r*_*long*_*time*_ = −1. The three rewards that have been formulated thus far are characterized as sparse rewards, and the task of training optimal policies using these sparse rewards poses significant challenges. Hence, an additional form of reward, known as a dense reward in contrast to a sparse reward, is introduced, which the Agent obtains at every timestep. The distance potential reward is formulated as the difference between the vehicle's distance from the endpoint at the present timestep and its distance from the endpoint at the preceding timestep as shown in Equation (15).


(15) $$\begin{array}{l} r_{potental}\\ =abs\left(loc_{end}\left(t\right)-loc_{vehicle}\left(t\right)\right)-abs\left(loc_{end}\left(t-1\right)-loc_{vehicle}\left(t-1\right)\right) \end{array}$$

This observation suggests that a positive reward is provided as the vehicle approaches the destination, while a negative reward is administered when the vehicle remains far from the destination. Likewise, it is desired for the autonomous vehicle to operate within a velocity range of 25–50 km per hour as shown in Equation (16).


(16) $$\begin{array}{l} r_{velocity}=\{\begin{array}{ll} v-25, & \text{if }v>25\\ v-25, & \text{if }25\leq v\leq 50\\ 50-v & \text{if }v>50 \end{array} \end{array}$$

The variable *v* represents the current velocity of the vehicle, with the rewards explicitly designed to meet the speed requirements of autonomous driving. Our overall reward is as follows:


(17) $$\begin{array}{l} r=c_{1}r_{success}+c_{2}r_{collision}+c_{3}r_{long\text{\_}time}+c_{4}r_{potental}+c_{5}r_{velocity} \end{array}$$

The reward coefficient, denoted as *c*_1_ through *c*_5_, is employed to achieve a balance among the individual rewards in Equation (17).

#### 5.3.3 DRLNDT training

Autonomous vehicles were trained interactively within the CARLA simulation environment, wherein they relied on a random policy to navigate the simulated world guided by rewards, the autonomous vehicle endeavors to execute the given navigation task successfully. Once the task either fails or succeeds, the episode of interaction finishes, prompting the start of a new episode. Simultaneously, the Agent evaluates and updates the value function and policy function by utilizing the information collected. The Agent will engage in interactions with the environment in accordance with the updated policy. The Agent acquires the optimal policy through engaging in ongoing interaction and ultimately achieves task completion.

During its travel, the autonomous vehicle gathers data pertaining to various states. The autonomous vehicle's motion induces modifications in the surrounding environment, resulting in the acquisition of new states. Initially, the autonomous vehicle acquires visual data and vector-based representations encompassing parameters such as velocity, position, and other relevant variables. Subsequently, the image input undergoes processing by a VAE in order to acquire latent states. These latent states are then merged with vector states to create a novel multimodal state. Subsequently, the state needs to undergo temporal serialization. In the academic domain of reinforcement learning, it is more appropriate to use the term “observation” instead of “state” to denote the concept being discussed. The conflation of observation and state occurs when the collected observation is indistinguishable from an actual state. As a result, in academic research, these two terms are often conflated for simplification. Nevertheless, in practical use, the data gathered by the Agent is often masked and lacks completeness. At the same time, the actual state remains concealed within the historical record of observations from before up until the present moment. The current state is derived from the sequence of observations using a transformer, allowing the Agent to acquire an improved policy. This characteristic represents the efficacy of our algorithm. We next need to historicize the state, or what can also be called the time serialization of the state. Serialization of the state adds a temporal dimension to the data. We consider the historical context of the present situation. The historical experience replay is a memory storage mechanism that stores a quintuple, denoted as $<s_{t}^{h},a_{t},r_{t},s_{t+1}^{h},done>$. In this quintuple, $s_{t}^{h}$ represents a set of historical states from *o*_*t*−*n*_ to *o*_*t*_, *a*_*t*_ represents the action taken at the current moment, *r*_*t*_ represents the reward acquired at the current moment, $s_{t+1}^{h}$ represents a set of historical states from *o*_*t*+1−*n*_ to *o*_*t*+1_, and *done* represents the end-of-episode marker. The approach employs a random sampling technique to select tuples from a historical experience replay. These selected tuples are then used to update both the value function and the policy function.

The training process adheres to the hyperparameters described in the Table 1. Specific hyperparameters are selected based on the optimum results reported in the paper, while others are determined through repeated training experiments to identify the most favorable findings.

**Table 1:** Hyperparameter list.

**Hyperparameters**	**Value**	**Description**
Camera resolution	640*640*3	Dimension of the input image
Dim_latent_vector	256	Dimension of the latent_vector
vae_lr	1.00E-04	VAE learning rate
Batch_size_vae	16	Batch size in VAE training
Train_data_size	65,536	Number of VAE training sets
*n*	50	Length of the history state sequence
Head_num	6	Number of heads in Transfomer
Dim_head	64	Dimension of head in Transfomer
Dim_mlp	512	Dimensions of MLPs in Transfomer
Buffer_size	65,536	Buffer size in SAC
lr	1e-3 1e-4	Learn rate in SAC
γ	0.99	Discount rate in SAC
batch_size	128	Batch size in SAC

### 5.4 Experimental results and analysis

**Figure 9:**
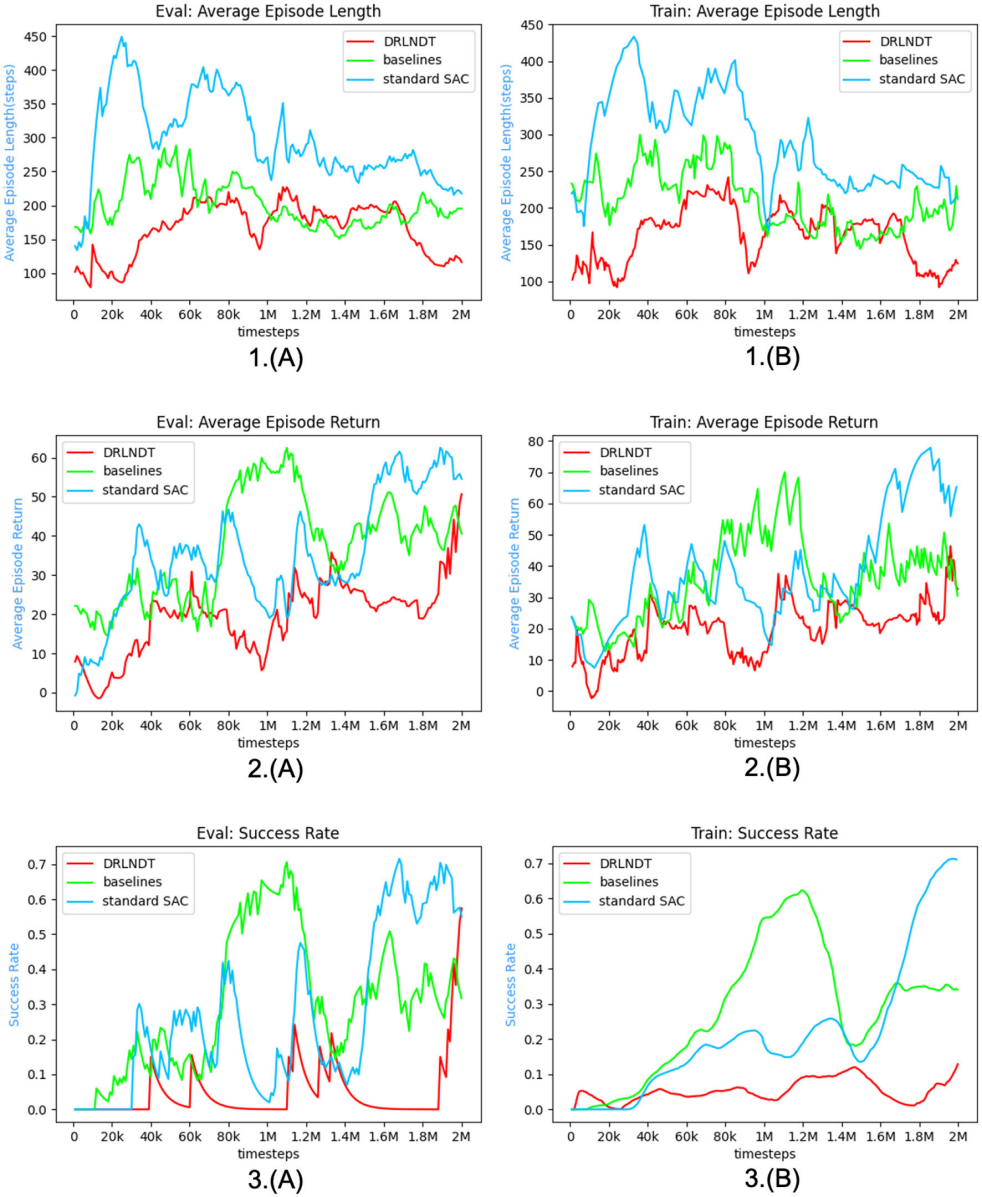
The figure presented illustrates the experimental outcomes of the DRLNDT method as compared to both the baseline and standard Soft Actor-Critic (SAC) algorithms throughout both the training and evaluation phases. The results provide evidence supporting the superiority of the DRLNDT algorithm over the other techniques. **(A)** Depicts the evaluation procedure, whereas **(B)** illustrates the training process. The chart's *x*-axis displays timesteps, while the *y*-axis shows *mean*_*len*, *mean*_*reward*, and *success*_*rate*.

**Figure 10:**
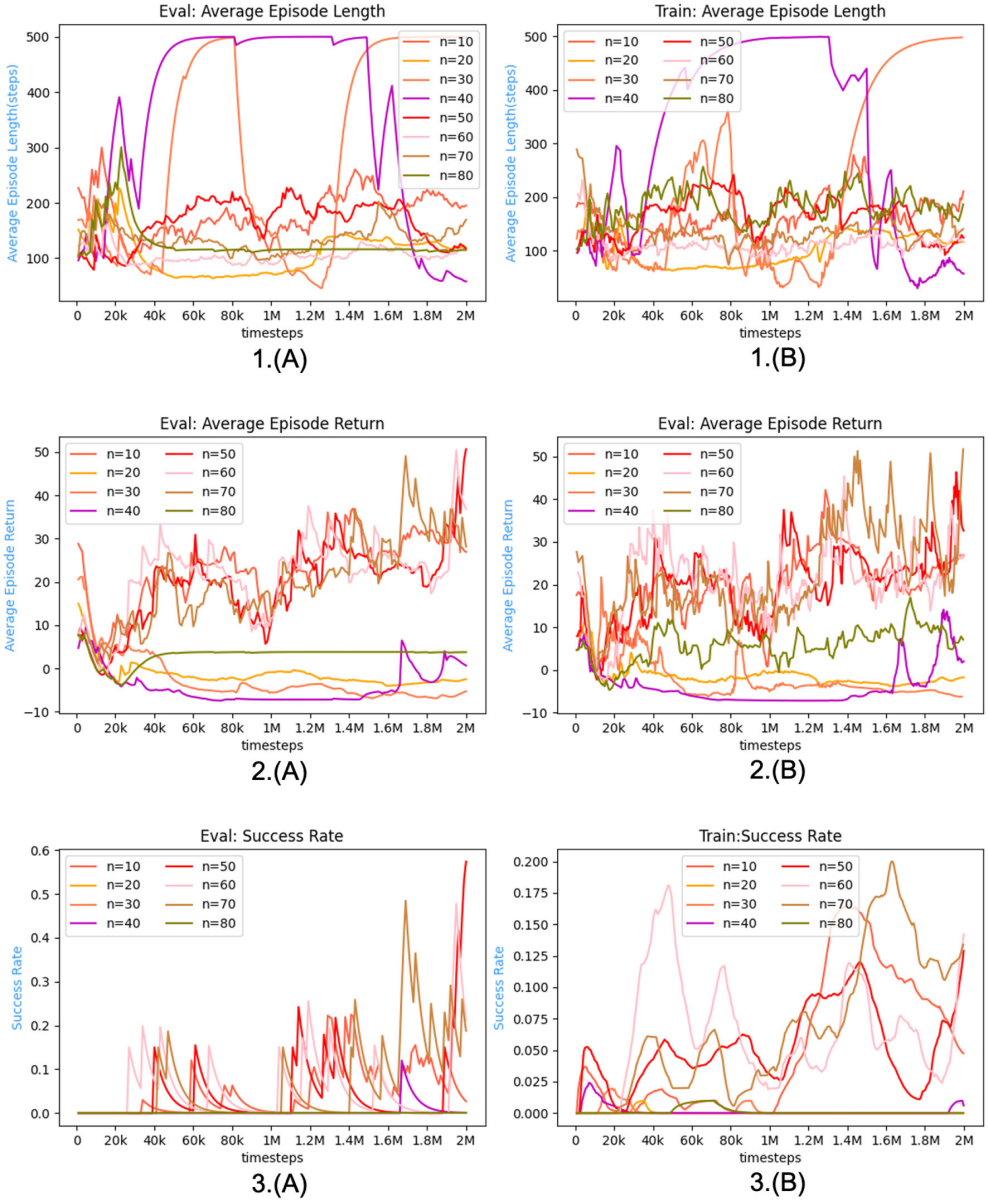
The figure illustrates the training and assessment outcomes obtained for various durations of history states. These history states are employed to elucidate the impact of varying lengths on the algorithms. **(A)** Depicts the evaluation procedure, whereas **(B)** illustrates the training process. The figure compares the results of the algorithms for time lengths ranging from *n* = 10 to *n* = 80.

**Figure 11:**
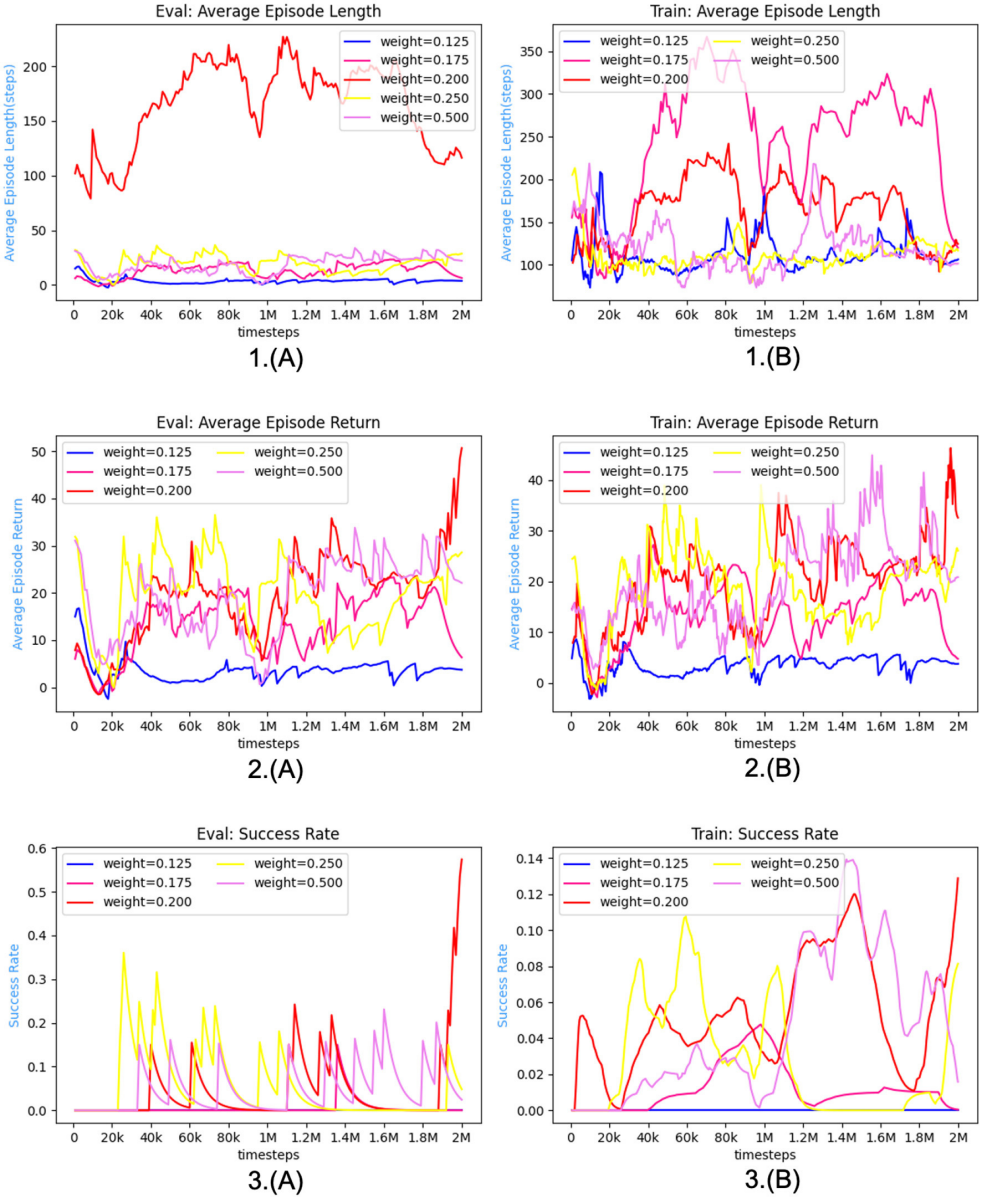
The figure illustrates the training and assessment outcomes obtained by varying the *potential*_*reward*_*weight* parameter. This analysis aims to investigate the impact of different *potential*_*reward*_*weight* values on the algorithm's performance. **(A)** Depicts the evaluation procedure, whereas **(B)** illustrates the training process. Specifically, the algorithmic results are compared across a range of *potential*_*reward*_*weight* values, ranging from 0.125 to 0.5.

The experiments primarily focus on evaluating the performance of our algorithm, DRLNDT, in achieving autonomous vehicle navigation from the starting point to the final destination. Additionally, we compare the experimental outcomes with those obtained using the baseline approach. The primary concept of the baseline technique involves utilizing a Recurrent Neural Network (RNN) to extract the latent state information from the previous states. Three evaluation standards are primarily utilized to assess the algorithm in comparison to the baseline algorithm during both the training and evaluation stages. The three evaluation metrics includeultra *mean*_*lens*, *mean*_*rewards*, and *success*_*rate*. The evaluation measure, indicated as *mean*_*lens*, quantifies the length of the sequence of the average episode, specifically measuring the average time spent at the conclusion of each episode. A negative correlation exists between the magnitude of the *mean*_*lens* and the time required to successfully perform the task. The evaluation metric *means*_*rewards* quantifies the average prizes acquired by the Agent after each episode on average. In the context of task completion, a more excellent value for the variable *means*_*rewards* signifies a heightened reward associated with the task. Consequently, this implies that the algorithm exhibits a greater level of effectiveness. The variable *success*_*rate* represents the proportion of successful episodes in achieving the task relative to the total number of episodes. It is considered the primary statistic for assessing the algorithm's efficacy. In our experimental study, we emphasize the investigation of reinforcement learning algorithms, while ignoring the assessment indicators pertaining to safety, comfort, and other relevant aspects of autonomous driving. There exist variations among these three indicators during the training and evaluation stages. During the training phase, our primary criterion for determining the end of training depended on the total number of time steps. During the evaluation phase, we analyzed the metrics statistically following 500 consecutive episode runs. Multiple sets of control experiments were conducted using the hyperparameters trained as listed in Table 1. The outcomes of these experiments are shown in Figures 9, 10, 11. The primary objective is to compare our DRLNDT method, the typical SAC algorithm, and the baseline algorithm to discern our algorithm's specific advantages while utilizing identical parameters. The data results are shown in Tables 2, 3.

**Table 2:** Comparison of experimental training processes.

**Policy**	**Train**			**Description**
	**Mean_length**	**Mean_reward**	**Success_rate**	
DRLNDT	160.0993	46.3408	0.128	Transformer + SAC
Baseline	192.1297	70.02924	0.623	RNN + SAC
Standard SAC	251.9917	77.8219	0.713	
DRLNDT-n-10	152.487	42.1571	0.1642	Historical state length *n* = 10
DRLNDT-n-20	77.869	13.363	0.0009	Historical state length *n* = 20
DRLNDT-n-30	75.24716	21.7094	0.0195	Historical state length *n* = 30
DRLNDT-n-40	85.8629	14.1125	0.024	Historical state length *n* = 40
DRLNDT-n-60	107.8153	45.3691	0.1809	Historical state length *n* = 60
DRLNDT-n-70	133.1698	51.724	0.1999	Historical state length *n* = 70
DRLNDT-n-80	179.1541	16.9834	0.00982	Historical state length *n* = 80
DRLNDT-w-0.125	105.7095	8.6156	0	Potential_reward_*w* = 0.125
DRLNDT-w-0.175	231.223	27.2584	0.0476	Potential_reward_*w* = 0.175
DRLNDT-w-0.25	107.2249	39.07225	0.1076	Potential_reward_*w* = 0.25
DRLNDT-w-0.5	111.2946	44.93245	0.139	Potential_reward_*w* = 0.5

**Table 3:** Comparison of experimental evaluation processes.

**Policy**	**Evaluation**			**Description**
	**Mean_length**	**Mean_reward**	**Success_rate**	
DRLNDT	110.055	80.3718	0.99502488	Transformer + SAC
Baseline	181.065	54.3497	0.59701493	RNN + SAC
Standard SAC	218.16	65.6080	0.7312	
DRLNDT-n-10	314.845	27.8312	0.398	Historical state length *n* = 10
DRLNDT-n-20	179.08	20.5407	0	Historical state length *n* = 20
DRLNDT-n-30	175.09	24.3238	0	Historical state length *n* = 30
DRLNDT-n-40	110.12	80.3325	0.99502488	Historical state length *n* = 40
DRLNDT-n-60	108.025	80.9850	0.995	Historical state length *n* = 60
DRLNDT-n-70	140.1	80.7300	0.995	Historical state length *n* = 70
DRLNDT-n-80	155.255	14.7500	0	Historical state length *n* = 80
DRLNDT-w-0.125	166.01	31.7600	0	Potential_reward_*w* = 0.125
DRLNDT-w-0.175	359.04	80.3810	0.99502	Potential_reward_*w* = 0.175
DRLNDT-w-0.25	110.015	80.5790	0.99502	Potential_reward_*w* = 0.25
DRLNDT-w-0.5	119.025	80.7813	0.99502	Potential_reward_*w* = 0.5
Modular Pipi	1113	83.0000	0.99	

The network was evaluated, and it was found that the DRLNDT method outperformed the Baseline algorithm and the Standard SAC algorithm. The hyperparameters were adjusted based on the values in Table 1. The evaluation process was divided into two stages - the training phase and the assessment phase. The results of the training and evaluatingphase are presented in Figure 9. We use three metrics to measure the algorithm's performance: *mean*_*len*, *mean*_*reward*, and *success*_*rate*. After training for 1,000 timesteps, the algorithm is evaluated ten times in a row. The performance metrics during the evaluation phase are calculated based on these assessments. It is intriguing to observe that DRLNDT performs superior to the other two measures in the *mean*_*len* metric. However, it notably exhibits inferior performance in the *success*_*rate* and *mean*_*reward* metrics compared to the other algorithms. We are profoundly contemplating this matter, which raises the question of whether our algorithm fails to enhance navigation performance. The provided response is incorrect; our algorithm demonstrates superiority over both algorithms. The primary metric of interest is the *success*_*rate*, which should be prioritized when evaluating the algorithm's ability to navigate successfully. The challenge of implementing navigation tasks using reinforcement learning algorithms arises from the intricate action and state spaces involved. Our algorithm performed exceptionally well and produced the model with the highest reward, which was used for subsequent testing. It is important to note that during the training phase and midway through it, the metrics obtained may not accurately represent the algorithm's overall performance but rather offer insights into the effectiveness of the training process. The stored model was deployed in the experimental environment for 200 testing episodes, during which various statistics such as mean episode length, mean episode reward, and success rate were computed and analyzed. The data results are presented in a tabular form, indicating that the evaluation steps depicted in the table differ from the outcomes illustrated in Figure 9. The table shows that the algorithm's performance strongly correlates with the observed results. Specifically, the algorithm has a success rate of 99.9%, which is higher than that of other algorithms. Additionally, our method achieves a higher average reward than alternative algorithms. Furthermore, the algorithm's *mean*_*len* metric is comparatively lower than others. Our algorithm has outperformed other algorithms, demonstrating its ability to learn the actual state from historical data. This method is particularly evident in our use of a transformer, which makes extracting the actual state from historical data more efficient than other algorithms. The transformer model can incorporate information from preceding and subsequent moments, thereby enabling the integration of a specific moment with its preceding and subsequent data. In contrast, recurrent neural networks (RNNs) can only establish relationships between a specific moment and its preceding moment. Consequently, the transformer model exhibits superior proficiency in integrating historical information compared to RNNs. The performance of the baseline algorithm was compared with that of the standard SAC algorithm, and it was found that the former showed a weaker effect than the latter. It could be due to the suboptimal performance of the RNN in extracting historical state information and its computational overhead. As a result, the standard SAC algorithm had a more pronounced effect than the baseline algorithm.

During the experiments, it was noticed that the variable *n*, which represents the duration of history, holds significant importance as it determines the temporal extent of the time series data. It is worth noting that the fixed time series were used for extracting the actual state instead of selecting variable time series. Choosing the correct length for a time series is critical for the algorithm. The algorithm may accurately capture underlying patterns if there is limited historical data. On the other hand, an excessive amount of historical data can cause the algorithm to consume too much memory and negatively impact its performance. As depicted in the Figure 10, we establish a range for the variable *n*, specifically from 10 to 80. Subsequently, we do a single training session at regular intervals of 10 units to ascertain the most favorable value for *n*. The figure demonstrates that the algorithm exhibits optimal performance at *n* = 50. Additionally, it is evident from the figure that at *n* = 50, the outcomes tend to reach a state of stability characterized by the absence of discernible fluctuations. Extending the training duration and period was found to be unfeasible as it resulted in oscillatory behavior and suboptimal results. When *n* ranges from 40 to 70, the success rates of the experimental outcomes are nearly identical, reaching up to 99%. The curves in the photos show a high degree of similarity. Furthermore, when evaluating the relationship between the average reward and the episode's duration, it becomes apparent that the optimal outcome is achieved when *n* equals 50. It is still possible to achieve good results even when *n* is ~50. It is worth mentioning that while evaluating the tests, there were minor differences in the speed of autonomous driving and a slight deviation from the ideal path regarding the traveled trajectory. To summarize, the Transformer-based method for extracting historical states performs better than other techniques. Additionally, the algorithm's effectiveness is closely associated with the length of the historical context. Specifically, the best results are achieved when *n* = 50. However, it should be noted that acceptable outcomes can still be attained within the range of the *n* = 50 parameter.

Generating reward weights can be a challenging task. The primary difficulty is determining the specific weights assigned to individual rewards, particularly the weight coefficient associated with *potential*_*reward*. An ablation experiment was conducted further to investigate the impact of the weight coefficient of *potential*_*reward*. The experiment consisted of five test groups, each with a different weight coefficient (0.125, 0.175, 0.2, 0.25, and 0.5). The weight coefficient was incrementally increased; the results are illustrated in Figure 11. The results of the experiments are summarized in the Tables 2, 3. The statistical analysis showed that setting the weight coefficient to 0.2 led to the best outcome. This finding highlights the importance of the hyperparameter *potential*_*reward* in guiding the autopilot task. Notably, *potential*_*reward* acts as a dense reward and is crucial in guiding the Agent to complete the task and acquire the optimal policy. This is particularly important because training the optimal policy for autopilot navigation is challenging due to the infinite-dimensional state and action space. In this context, *potential*_*reward* is the only guiding factor for the Agent to complete the navigation task instead of relying on sparse rewards Through the evaluation phase, it was observed that increasing the weight of *potential*_*reward* leads to favorable outcomes for the algorithm. However, during the training phase, it was discovered that large weights hinder the learning process, causing the algorithm to converge locally. It is demonstrated by the autonomous vehicle repeatedly circling in place or remaining stationary, resulting in its inability to reach the optimal policy. In the middle of the training, we thought that we would not be able to train a good policy, but after saving the model with the maximum reward, we found that it still showed good results with a success rate of 99%, which we did not expect in our experiments. Therefore, the weight of *potential*_*reward* should be a manageable size, and after comparing the length of the episodes and the average reward, a weight of 0.2 was the best choice.

Our DRLNDT algorithm has outperformed both the baseline and other algorithms based on the comparison experiments conducted. This algorithm effectively guides an autonomous vehicle from its initial to the termination position while simultaneously learning the optimal policy. During the evaluation phase, we saved the model with the highest rewards and utilized it for assessment. After conducting 200 consecutive testing episodes, we achieved a notable success rate of 99%. Furthermore, a comparison was made between the traditional modular approach and our algorithm. The results indicate that the traditional modular (Paden et al., 2016) algorithm does not yield superior outcomes, primarily due to incorrect routing during the navigation phase, resulting in erroneous trajectory routes. It highlights the advantage of our policy, as the interdependence of modular navigation tasks can lead to failure or reduced effectiveness when routing errors occur. The experimental results indicate that the DRLNDT approach is a successful solution for resolving the problem of state unobservability in the POMDP model. This method conceals the current state by incorporating it into the historical state, utilizing the Transformer model. It is worth noting that the Transformer model performs better than the LSTM-based RNN algorithm in terms of effectiveness.

We compare the computational performance of the methods and evaluate their performance in the evaluation phase based on response time. The Table 4 displays the mean response time of each method during the evaluation portion of the examination, which is measured in milliseconds. The computation time is determined by executing the algorithms in the Python framework. The response time indicates the algorithm's decision-making capability, which influences its capacity to handle unforeseen circumstances. A faster response time can enhance the safety of autonomous driving during emergencies. The classic modular method has the quickest reaction time, while the DRLNDT algorithm has the longest response time among the other algorithms. The 7 ms difference in reaction time between the two frameworks is insignificant for practical purposes, especially considering that the response time in the C++ framework is much shorter. The DRLNDT algorithm demonstrates superior performance compared to other algorithms across all metrics. The algorithm we are comparing is a simplified version that relies on a detailed map with pre-marked data on intersections, traffic lights, and obstacles for navigation. The modular approach is aware of the knowledge of surrounding barriers beforehand, but in real-world scenarios, it must be combined with sensing, localization, routing modules, and so forth. The navigation process in actual applications will be delayed by the inclusion of a sensor module, localization module, and other components, leading to increased response time. Traditional approaches are slower in resolving complicated situations due to the rise in constraints. Our method relies on an end-to-end reinforcement learning algorithm and does not impact response time despite the environment's complexity. The Transformer model's benefit lies in its ability to handle each location in the sequence simultaneously, leading to a notable enhancement in training speed and performance. Its self-attentive mechanism allows it to capture both long-distance dependencies and local structural and positional information in the sequence simultaneously. As a result, by incorporating a transformer into our DRLNDT algorithm, historical information can be incorporated to extract the actual state, thereby enhancing the algorithm's decision-making capability, resolving the issue of unobservable states in the POMDP model, and producing an outcome that surpasses that of the baseline algorithm. The intricate design of the transformer model results in a high requirement for computer resources during training and inference, impacting the reaction time during actual deployment. The VAE in our DRLNDT algorithm retrieves latent states from the source image to preserve its features while lowering the dimensionality of other images. Our technique reduces the amount of parameters in the Transformer model. Our real-world experiments showed that the model had fewer than 100 million parameters, making it suitable for deployment in real-world settings. Our approach, being end-to-end based, optimizes the hardware performance dedicated to decision-making. The response time remains unaffected by the heavy demand for processing resources.

**Table 4:** Experimental evaluation process on response time comparison.

**Policy**	**Response_time**	**Description**
DRLNDT	7.4250	Transformer + SAC
Baseline	2.8370	RNN + SAC
Standard SAC	2.7200	
DRLNDT-n-10	7.1830	Historical state length *n* = 10
DRLNDT-n-20	8.1440	Historical state length *n* = 20
DRLNDT-n-30	5.7540	Historical state length *n* = 30
DRLNDT-n-40	7.4040	Historical state length *n* = 40
DRLNDT-n-60	7.4030	Historical state length *n* = 60
DRLNDT-n-70	7.4046	Historical state length *n* = 70
DRLNDT-n-80	7.7540	Historical state length *n* = 80
DRLNDT-w-0.125	7.4350	Potential_reward_*w* = 0.125
DRLNDT-w-0.175	7.4130	Potential_reward_*w* = 0.175
DRLNDT-w-0.25	7.5120	Potential_reward_*w* = 0.25
DRLNDT-w-0.5	7.3780	Potential_reward_*w* = 0.5
Modular Pipi	1.3000	

## 6 Conclusion and future

This paper provides a comprehensive overview of the DRLNDT algorithm for autonomous vehicle navigation. The paper also discusses the experimental outcomes obtained by implementing our algorithm on the CARLA platform. The results substantiate the superiority of our approach over the baseline approach. The available evidence adequately supports the efficacy of our approach. We have employed the Transformer model in our research to address the issue of incomplete observation of the state in POMDP due to sensor occlusion or noise in autonomous driving. We aim to learn the real state from the historical state with the help of this model. We have successfully developed an optimal policy for autonomous driving, enabling the vehicle to navigate from the starting to the termination position.

Our algorithm has achieved an impressive 99% success rate in a complex state and action space task using only high-quality monocular images without any prior knowledge of high-precision maps, routing, or surrounding environment information. This outcome is a testament to the effectiveness of our optimal policy.

Despite our best efforts, implementing our system in actual vehicles has proven to be a challenging task. While our problem is considered highly challenging in the field of reinforcement learning, it is relatively straightforward when compared to the complexities of real-world self-driving navigation tasks. The current system needs to be more effective in handling intricate navigation challenges, including randomized starting and ending positions, which require additional algorithmic improvements. Based on our analysis, we have identified that the need for appropriate incentives designed by humans is the primary cause of the issue. To overcome this challenge, we plan to focus our future research on reward design for self-driving navigation tasks. We will use the active preference learning approach to gain knowledge about the reward weights associated with complex human needs. Additionally, we intend to explore areas such as learning the reward function from expert data through inverse learning. These two focal points will be the primary areas of investigation for our future research projects.

## Data availability statement

The original contributions presented in the study are included in the article/supplementary material, further inquiries can be directed to the corresponding author.

## Author contributions

LG: Investigation, Methodology, Software, Writing – original draft, Writing – review & editing. XZ: Resources, Supervision, Writing – review & editing. YW: Data curation, Funding acquisition, Resources, Writing – review & editing. YL: Writing – review & editing.

## References

[B1] Andrychowicz M. Raichuk A. Stańczyk P. Orsini M. Girgin S. Marinier R.. (2020). What matters in on-policy reinforcement learning? A large-scale empirical study. arXiv. [Preprint]. 10.48550/arXiv.2006.05990

[B2] Anzalone L. Barra P. Barra S. Castiglione A. Nappi M. (2022). An end-to-end curriculum learning approach for autonomous driving scenarios. IEEE Trans. Intell. Transp. Syst. 23, 19817–19826. 10.1109/TITS.2022.3160673

[B3] Arulkumaran K. Deisenroth M. P. Brundage M. Bharath A. A. (2017). A brief survey of deep reinforcement learning. arXiv. [Preprint]. 10.48550/arXiv.1708.05866

[B4] Chen J. Yuan B. Tomizuka M. (2019). “Model-free deep reinforcement learning for urban autonomous driving,” in 2019 IEEE Intelligent Transportation Systems Conference (ITSC) (Aucklan: IEEE), 2765–2771. 10.1109/ITSC.2019.8917306

[B5] Chen L. Lu K. Rajeswaran A. Lee K. Grover A. Laskin M.. (2021). Decision transformer: reinforcement learning via sequence modeling. Adv. Neural Inf. Process. Syst 34, 15084–15097.

[B6] Choromanski K. Likhosherstov V. Dohan D. Song X. Gane A. Sarlos T.. (2020). Rethinking attention with performers. arXiv. [Preprint]. 10.48550/arXiv.2009.14794

[B7] Ding M. Zhou C. Yang H. Tang J. (2020). Cogltx: applying bert to long texts. Adv. Neural Inf. Process. Syst. 33, 12792–12804.

[B8] Dosovitskiy A. Beyer L. Kolesnikov A. Weissenborn D. Zhai X. Unterthiner T.. (2020). An image is worth 16x16 words: transformers for image recognition at scale. arXiv. [Preprint]. 10.48550/arXiv.2010.11929

[B9] Dosovitskiy A. Ros G. Codevilla F. Lopez A. Koltun V. (2017). “CARLA: an open urban driving simulator,” in Proceedings of the 1st Annual Conference on Robot Learning, eds S. Levine, V. Vanhoucke, and K. Goldberg (Mountain View, CA: PMLR), 1–16. Available online at: http://proceedings.mlr.press/v78/dosovitskiy17a/dosovitskiy17a.pdf

[B10] Ghosh D. Rahme J. Kumar A. Zhang A. Adams R. P. Levine S.. (2021). Why generalization in rl is difficult: epistemic pomdps and implicit partial observability. Adv. Neural Inf. Process. Syst. 34, 25502–25515.

[B11] González D. Pérez J. Milanés V. Nashashibi F. (2015). A review of motion planning techniques for automated vehicles. IEEE Trans. Intell. Transp. Syst. 17, 1135–1145. 10.1109/TITS.2015.2498841

[B12] Haarnoja T. Zhou A. Hartikainen K. Tucker G. Ha S. Tan J.. (2018). Soft actor-critic algorithms and applications. arXiv. [Preprint]. 10.48550/arXiv.1812.05905

[B13] Hausknecht M. Stone P. (2015). “Deep recurrent q-learning for partially observable MDPS,” in 2015 AAAI Fall Symposium Series.

[B14] Heess N. Hunt J. J. Lillicrap T. P. Silver D. (2015). Memory-based control with recurrent neural networks. arXiv. [Preprint]. 10.48550/arXiv.1512.04455

[B15] Igl M. Zintgraf L. Le T. A. Wood F. Whiteson S. (2018). Deep variational reinforcement learning for pomdps. Proc. 35th Intl. Conf. Machine Learn. Proc. Mach. Learn. Res. 80, 2117–2126.

[B16] Janner M. Li Q. Levine S. (2021). Offline reinforcement learning as one big sequence modeling problem. Adv. Neural Inf. Process. Syst. 34, 1273–1286.

[B17] Kaelbling L. P. Littman M. L. Cassandra A. R. (1998). Planning and acting in partially observable stochastic domains. Artif. Intell. 101, 99–134. 10.1016/S0004-3702(98)00023-X

[B18] Kendall A. Hawke J. Janz D. Mazur P. Reda D. Allen J.-M.. (2019). “Learning to drive in a day,” in 2019 International Conference on Robotics and Automation (ICRA) (IEEE), 8248–8254. 10.1109/ICRA.2019.8793742

[B19] Kiran B. R. Sobh I. Talpaert V. Mannion P. Al Sallab A. A. Yogamani S.. (2021). Deep reinforcement learning for autonomous driving: a survey. IEEE Trans. Intell. Transp. Syst. 23, 4909–4926. 10.1109/TITS.2021.3054625

[B20] Liang X. Wang T. Yang L. Xing E. (2018). “CIRL: controllable imitative reinforcement learning for vision-based self-driving,” in Proceedings of the European Conference on Computer Vision (ECCV) (Cham: Springer), 584–599. 10.1007/978-3-030-01234-2_36

[B21] Loaiza-Ganem G. Cunningham J. P. (2019). The continuous Bernoulli: fixing a pervasive error in variational autoencoders. Adv. Neural Inf. Process. Syst. 32.

[B22] Mnih V. Kavukcuoglu K. Silver D. Graves A. Antonoglou I. Wierstra D.. (2013). Playing atari with deep reinforcement learning. arXiv. [Preprint]. 10.48550/arXiv.1312.5602

[B23] Mnih V. Kavukcuoglu K. Silver D. Rusu A. A. Veness J. Bellemare M. G.. (2015). Human-level control through deep reinforcement learning. Nature 518, 529–533. 10.1038/nature14236 25719670

[B24] Morales E. F. Murrieta-Cid R. Becerra I. Esquivel-Basaldua M. A. (2021). A survey on deep learning and deep reinforcement learning in robotics with a tutorial on deep reinforcement learning. Intell. Serv. Robot. 14, 773–805. 10.1007/s11370-021-00398-z

[B25] Nair A. Srinivasan P. Blackwell S. Alcicek C. Fearon R. De Maria S.. (2015). Massively parallel methods for deep reinforcement learning. arXiv. [Preprint]. 10.48550/arXiv.1507.04296

[B26] Ozturk A. Gunel M. B. Dagdanov R. Vural M. E. Yurdakul F. Dal M.. (2021). “Investigating value of curriculum reinforcement learning in autonomous driving under diverse road and weather conditions,” in 2021 IEEE Intelligent Vehicles Symposium Workshops (IV Workshops) (Nagoya: IEEE), 358–363. 10.1109/IVWorkshops54471.2021.9669203

[B27] Paden B. Čáp M. Yong S. Z. Yershov D. Frazzoli E. (2016). A survey of motion planning and control techniques for self-driving urban vehicles. IEEE Trans. Intell. Veh. 1, 33–55. 10.1109/TIV.2016.2578706

[B28] Parisotto E. Song F. Rae J. Pascanu R. Gulcehre C. Jayakumar S.. (2020). “Stabilizing transformers for reinforcement learning,” in International Conference on Machine Learning PMLR (Vienna: ACM), 7487–7498.

[B29] Parmar N. Vaswani A. Uszkoreit J. Kaiser L. Shazeer N. Ku A.. (2018). “Image transformer,” in Proceedings of the 35th International Conference on Machine Learning, volume 80 of Proceedings of Machine Learning Research, eds J. Dy, and A. Krause (Stockholm: PMLR), 4055–4064.

[B30] Puterman M. L. (2014). Markov Decision Processes: Discrete Stochastic Dynamic Programming. Hoboken, NJ: John Wiley and Sons.

[B31] Silver D. Lever G. Heess N. Degris T. Wierstra D. Riedmiller M.. (2014). “Deterministic policy gradient algorithms,” in International Conference on Machine Learning (PMLR), 387–395.

[B32] Tamar A. Wu Y. Thomas G. Levine S. Abbeel P. (2016). Value iteration networks. Adv. Neural Inf. Process. Syst. 29. 10.24963/ijcai.2017/700

[B33] Vaswani A. Shazeer N. Parmar N. Uszkoreit J. Jones L. Gomez A. N.. (2017). Attention is all you need. Adv. Neural Inf. Process. Syst. 30.

[B34] Wang S. Li B. Z. Khabsa M. Fang H. Ma H. (2020). Linformer: self-attention with linear complexity. arXiv. [Preprint]. 10.48550/arXiv.2006.04768

[B35] Watkins C. J. Dayan P. (1992). Q-learning. Mach. Learn. 8, 279–292. 10.1007/BF00992698

[B36] Wei R. Garcia C. El-Sayed A. Peterson V. Mahmood A. (2020). Variations in variational autoencoders-a comparative evaluation. IEEE Access 8, 153651–153670. 10.1109/ACCESS.2020.3018151

[B37] Ye F. Zhang S. Wang P. Chan C.-Y. (2021). “A survey of deep reinforcement learning algorithms for motion planning and control of autonomous vehicles,” in 2021 IEEE Intelligent Vehicles Symposium (IV) (Nagoya: IEEE), 1073–1080. 10.1109/IV48863.2021.9575880

[B38] Yeom K. (2022). Deep reinforcement learning based autonomous driving with collision free for mobile robots. Int. J. Mech. Eng. Robot. Res. 11, 338–344. 10.18178/ijmerr.11.5.338-344

[B39] Yoo D. Park S. Lee J.-Y. Paek A. S. So Kweon I. (2015). “Attentionnet: aggregating weak directions for accurate object detection,” in Proceedings of the IEEE International Conference on Computer Vision (Santiago: IEEE), 2659–2667. 10.1109/ICCV.2015.305

[B40] Yu Y. Si X. Hu C. Zhang J. (2019). A review of recurrent neural networks: LSTM cells and network architectures. Neural Comput. 31, 1235–1270. 10.1162/neco_a_01199 31113301

[B41] Zhu P. Li X. Poupart P. Miao G. (2017). On improving deep reinforcement learning for pomdps. arXiv. [Preprint]. 10.48550/arXiv.1704.07978

